# Novel 1,2,3-triazole–thiophene hybrids: synthesis, anti-MRSA/MSSA activity, antioxidant potential, and acetylcholinesterase ınhibition supported by molecular docking

**DOI:** 10.1007/s00210-026-05402-w

**Published:** 2026-05-05

**Authors:** Mehmet Erşatır

**Affiliations:** Department of Chemistry, Faculty of Art and Science, Cukurova University, Adana, 01330 Türkiye

**Keywords:** 1,2,3-triazole–thiophene hybrids, Multicomponent reaction synthesis, Antimicrobial activity, Methicillin-resistant *Staphylococcus aureus*, Acetylcholinesterase inhibition, Molecular docking

## Abstract

The discovery of single-molecule therapeutics capable of simultaneously addressing antibiotic resistance and neurodegeneration represents a critical unmet need in modern medicinal chemistry. Herein, I report the rational design and efficient one-pot synthesis of a novel class of 1,2,3-triazole–thiophene hybrids (2a-2e) as dual-target bioactive agents. This streamlined synthetic approach afforded the target compounds in good yields, with substituent effects playing a decisive role in reaction efficiency. Biological evaluation revealed a pronounced enhancement in antibacterial activity, with several compounds exhibiting potent efficacy against both methicillin-susceptible and methicillin-resistant *Staphylococcus aureus* (MRSA). In particular, compounds 2d and 2e demonstrated remarkable potency, achieving minimum inhibitory concentration (MIC) values as low as 2 µg/mL against MRSA. Structure–activity relationship analysis identified para-halogen substitution as a key determinant of antibacterial performance. Beyond their antibacterial effects, the hybrids displayed significant acetylcholinesterase (AChE) inhibitory activity, with compounds 2c (IC_50_ = 25.914 ± 0.061 µg/mL) and 2d (IC_50_ = 27.664 ± 0.238 µg/mL) emerging as the most active analogues. Complementary antioxidant assays further underscored the multifunctional character of these scaffolds. Molecular docking studies provided mechanistic support for the experimental findings, revealing favorable binding modes within the AChE active site. Collectively, this work establishes 1,2,3-triazole–thiophene hybrids as a versatile and previously underexplored scaffold for dual-target drug design. The identified lead compounds (2c-2e) demonstrate compelling potential for further optimization toward integrated therapeutic strategies against antibiotic-resistant infections and neurodegenerative disorders.

**Supplementary Information:**

The online version contains supplementary material available at 10.1007/s00210-026-05402-w.

## Introduction

Antibacterial resistance has emerged as one of the most critical global health threats, substantially compromising the efficacy of conventional antibiotic therapies. Among resistant pathogens, *Staphylococcus aureus* is a leading causative agent of both community- and hospital-acquired infections, ranging from mild skin infections to life-threatening conditions such as pneumonia, bacteremia, and endocarditis. In particular, methicillin-resistant *S. aureus* (MRSA) strains have acquired resistance to multiple β-lactam antibiotics, rendering treatment increasingly challenging and contributing to elevated morbidity and mortality rates. Although methicillin-susceptible *S. aureus* (MSSA) strains generally remain more responsive to available antibiotics, their capacity to rapidly develop resistance continues to represent a significant clinical concern (Yazici et al. 2024). Accordingly, the identification and development of novel antibacterial agents effective against both MSSA and MRSA remain an urgent priority in antimicrobial drug discovery (Demirbag et al. 2025; Yıldırım et al. 2025, 2026a; Çimentepe et al. 2026; Öztürk et al. 2026; Yildirim et al. 2026d).

Alzheimer’s disease (AD) is a progressive and irreversible neurodegenerative disorder and a major global health burden associated with aging populations. It is characterized by cognitive decline, including impairments in memory, learning, and executive functions, ultimately leading to dementia and loss of independence. At the molecular level, AD is defined by β-amyloid plaque accumulation and tau protein hyperphosphorylation, which disrupt synaptic function, trigger oxidative stress and neuroinflammation, and result in neuronal loss, particularly in the hippocampus and cerebral cortex. As the disease progresses, widespread neurodegeneration leads to brain atrophy and severe impairment of cognitive and physiological functions. In addition to its clinical impact, AD imposes a substantial socioeconomic burden, with global dementia-related costs exceeding USD 1 trillion annually and prevalence steadily increasing due to population aging. Mechanistically, the cholinergic hypothesis attributes cognitive deficits to reduced acetylcholine levels caused by cholinergic neuron degeneration, making acetylcholinesterase (AChE) a key therapeutic target. However, currently approved AChE inhibitors provide only limited symptomatic benefit and are often associated with adverse effects, underscoring the need for more effective and safer therapeutic strategies (Poyraz et al. 2023, 2024; Çimentepe et al. 2026; Öztürk et al. 2026; Yıldırım et al. 2026a).

In recent years, heterocyclic compounds have attracted considerable attention in drug discovery due to their structural diversity and wide range of biological activities (Akgul et al. 2025; Yilmaz et al. 2026; Yıldırım et al. 2026b, c). Among these, triazole derivatives have emerged as particularly valuable scaffolds in medicinal chemistry (Liu 2019; Kumar and Saroha 2022; Saroha 2022; Wang et al. 2024a, 2025; Yan et al. 2024; Guo and He 2025; Kumar et al. 2025a; Li et al. 2025; Guo et al. 2026). These compounds exhibit a broad spectrum of pharmacological properties, including anticancer (Raju et al. 2016; Mohamed et al. 2023; Irrou et al. 2024; Kumar et al. 2024a), antitubercular (Dheer et al. 2017), antiviral (Esteves et al. 2025), anti-inflammatory (Dharavath et al. 2020; Wang et al. 2024a, b), antileishmanial (Seck et al. 2023), antimicrobial (Sumangala et al. 2010; Wang et al. 2010; Raju et al. 2016; El-sayed et al. 2019; Gandham et al. 2022; Yadav et al. 2022; Kumar et al. 2024b, 2025b; Abualnaja et al. 2025), and anti-Alzheimer activities (Lu et al. 2020; Çesme et al. 2024). Their versatility stems from their unique physicochemical properties and ability to interact effectively with biological macromolecules (Haider et al. 2014; Bonandi et al. 2017; Dheer et al. 2017; Almaz 2023; Khan et al. 2023).

An important strategy in modern medicinal chemistry involves the design of hybrid molecules that combine two or more biologically active pharmacophores within a single framework. This hybridization approach can enhance biological activity, improve selectivity, and reduce toxicity by exploiting synergistic interactions between different structural motifs. Numerous studies have demonstrated that hybrid compounds incorporating the 1,2,3-triazole scaffold exhibit significantly enhanced biological activities compared to their parent structures. For example, indole/phthalimide/oxadiazole-based hybrids have been reported to show potent anticancer activity (Mohamed et al. 2023). Thioquinoxaline-containing hybrids demonstrate promising antibacterial properties (Keivanloo et al. 2020), while benzoxepin-derived molecules exhibit notable antimicrobial activity (Gandham et al. 2022). Coumarin-based hybrid structures have been widely investigated for their antioxidant, anti-inflammatory, and antimicrobial effects (Dharavath et al. 2020). Similarly, sulfonamide-containing hybrids have shown a broad range of pharmacological activities, including antioxidant, anticancer, anti-Alzheimer, antibacterial, and antifungal properties (Wang et al. 2010). In addition, thiophene-containing derivatives have attracted attention due to their strong antimicrobial and antiviral potential (Abualnaja et al. 2025). Combining the pharmacological advantages of the 1,2,3-triazole ring with the bioactive properties of thiophene moieties represents a promising strategy for the development of novel therapeutic agents (Bonandi et al. 2017; Dheer et al. 2017; Almaz 2023; Khan et al. 2023).

Accordingly, the combination of 1,2,3-triazole and thiophene moieties represents a promising approach for the development of multifunctional therapeutic agents. Based on this rationale, the present study reports the design, synthesis, and biological evaluation of a novel series of 1,2,3-triazole–thiophene hybrid compounds. The synthesized derivatives were evaluated for their antibacterial activity against MSSA and MRSA strains, antioxidant potential using DPPH and ABTS assays, and acetylcholinesterase inhibitory activity. Furthermore, molecular docking studies were performed to elucidate the binding interactions of the compounds with the AChE active site at the molecular level. This integrated approach aims to provide insight into structure–activity relationships and support the development of new multifunctional drug candidates with potential applications in the treatment of Alzheimer’s disease and bacterial infections.

## Materials and methods

### Instruments and chemicals

All chemicals and solvents used in the experimental procedures were commercially obtained from Aldrich or Merck and used without further purification unless otherwise stated. Melting points (MP) were determined using a capillary melting point apparatus, specifically the Electrothermal 9100 model, and reported without correction. IR spectra were recorded in the range of 4000–400 cm^−1^ using a Perkin Elmer 55148 FT-IR spectrometer. ^1^H and ^13^C NMR spectra were acquired in DMSO_4_-*d*_*6*_ or CDCl_3_ with tetramethylsilane (TMS) as an internal standard using a Bruker 600 MHz NMR spectrometer. Elemental analyses (C, H, N) were performed on a Thermo Flash 2000 Organic Elemental Analyzer (CHN). Absorbance was measured at 517 nm using a Shimadzu UV-1800 spectrophotometer.

### Synthesis of 1-[4-(substituted phenyl)]−4-acetyl-5-methyl-1,2,3-triazoles (1a–1e)

**Scheme 1:**
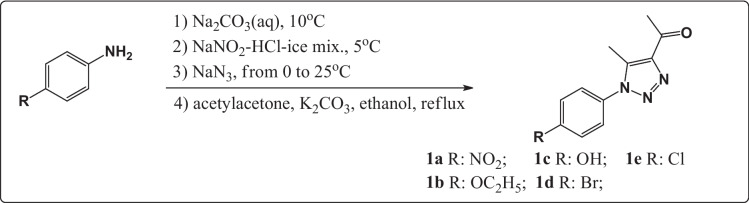
Synthesis of 1-[4-(substituted phenyl)]−4-acetyl-5-methyl-1,2,3-triazoles (1a–1e)

To a 100 mL two-necked round-bottom flask, p-substituted aniline (10 mmol) and sodium carbonate (5 mmol) were added and stirred in an ice bath. Separately, NaNO_2_ (10 mmol) was dissolved in 2 mL of distilled water, and concentrated HCl (2 mL) was mixed with 2 mL of distilled water. This solution was added dropwise to the flask while carefully maintaining temperature to prevent sudden increases. Sodium azide (NaN_3_, 10 mmol) was then added, and the mixture was allowed to reach ambient temperature (~ 25 °C) spontaneously. Upon reaching the desired temperature, acetylacetone (10 mmol), potassium carbonate (15 mmol), and 15 mL of ethanol were added, and the mixture was heated under reflux for 1–4 h (Scheme 1). Reaction progress was monitored by thin-layer chromatography (TLC). At the end of the reaction, the mixture was cooled to room temperature and poured into an ice–water mixture with stirring. The resulting precipitate was filtered, washed with cold water, and recrystallized from absolute ethanol.

#### 1-(5-methyl-1-(4-nitrophenyl)−1H-1,2,3-triazol-4-yl)ethanone (1a) (Kamalraj et al. 2008)

Yellow solid; mp:148–149 °C; yield: 2.438 g (%99.02); Rf = 0.41 (n-hexane/ethyl acetate, 2:3); υ_max_ (KBr) 1687 cm^−1^ (C = O stretching), 1604 cm^−1^ (N = N stretching), 1349 cm^−1^ (Ar-NO_2_ stretching), 3061 cm^−1^ (aromatic C-H stretching); δ_H_ (600 MHz, DMSO_4_-*d*_*6*_) 7.94 (d, *J* = 9.6 Hz, 2H, Ar–*H*), 6.61 (d, *J* = 9.6 Hz, 2H, Ar–*H*), 2.51 (s, 3H, C*H*_3_), 1.60 (s, 3H, C*H*_3_); δ_C_ (150 MHz, DMSO_4_-d_*6*_) 189.34 (-*C* = O), 156.23 (Ar–*C*-NO_2_), 146.66 (Ar–*C*-N), 136.06 (H_3_C-*C* = C-), 130.28(H_3_C-C = *C*-), 126.84 (Ar–*C*), 112.85 (Ar–*C*), 26.44 (H_3_*C*-C = O), 10.27 (*C*H_3_); Anal. Calcd. for C_11_H_10_N_4_O_3_: C, 53.66; H, 4.09; N, 22.75. Found: C, 53.75; H, 4.08; N, 22.70.

#### 1-(1-(4-ethoxyphenyl)−5-methyl-1H-1,2,3-triazol-4-yl)ethanone (1b)

Brown solid; mp:120–121 °C; yield: 1.300 g (%53.00); Rf = 0.57 (n-hexane/ethyl acetate, 2:3); υ_max_ (KBr) 1691 cm^−1^ (C = O stretching), 1512 cm^−1^ (N = N stretching), 3030 cm^−1^ (aromatic C-H stretching); δ_H_ (600 MHz, CDCl_3_) 7.31 (d, *J* = 12.0 Hz, 2H, Ar–*H*), 6.88 (d, *J* = 12.0 Hz, 2H, Ar–*H*), 4.04 (q, *J* = 12.6 Hz, 2H, -C*H*_2_), 2.59 (s, 3H, C*H*_3_), 2.29 (s, 3H, C*H*_3_), 1.41 (t, *J* = 12.6 Hz, 3H, -C*H*_3_); δ_C_ (150 MHz, CDCl_3_) 197.55 (-*C* = O), 157.41 (Ar–*C*-OC_2_H_5_), 134.91 (Ar–*C*-N), 132.61 (H_3_C-*C* = C-), 124.29 (H_3_C-C = *C*-), 117.64 (Ar–*C*), 115.10 (Ar–*C*), 63.74 (-O*C*H_2_), 31.57 (H_3_*C*-C = O), 26.64 (-OCH_2_*C*H_3_), 14.87 (*C*H_3_); Anal. Calcd. for C_13_H_15_N_3_O_2_: C, 63.66; H, 6.16; N, 17.13. Found: C, 63.77; H, 6.14; N, 17.09.

#### 1-(1-(4-hydroxyphenyl)−5-methyl-1H-1,2,3-triazol-4-yl)ethanone (1c)

Black solid; mp:169–170 °C; yield: 1.607 g (%73.98); Rf = 0.57 (n-hexane/ethyl acetate, 2:3); υ_max_ (KBr) 1693 cm^−1^ (C = O stretching), 1510 cm^−1^ (N = N stretching), 3013 cm^−1^ (aromatic C-H stretching), 3354 cm^−1^ (O–H stretching); δ_H_ (600 MHz, DMSO_4_-*d*_*6*_) 7.94 (d, *J* = 9.6 Hz, 2H, Ar–*H*), 6.61 (d, *J* = 9.6 Hz, 2H, Ar–*H*), 2.51 (s, 3H, C*H*_3_), 1.60 (s, 3H, C*H*_3_); δ_C_ (150 MHz, DMSO_4_-d_*6*_) 190.28 (-*C* = O), 155.68 (Ar–*C*-OH), 135.89 (H_3_C-*C* = C-), 130.82 (H_3_C-C = *C*-), 126.82 (Ar–*C*-N), 122.63 (Ar–*C*), 117.40 (Ar–*C*), 25.81 (H_3_*C*-C = O), 13.47 (*C*H_3_); Anal. Calcd. for C_11_H_11_N_3_O_2_: C, 60.82; H, 5.10; N, 19.34. Found: C, 60.91; H, 5.09; N, 19.31.

#### 1-(1-(4-bromophenyl)−5-methyl-1H-1,2,3-triazol-4-yl)ethanone (1d) (Kamalraj et al. 2008)

Orange solid; mp:120–121 °C; yield: 2.773 g (%98.99); Rf = 0.43 (n-hexane/ethyl acetate, 2:3); υ_max_ (KBr) 1680 cm^−1^ (C = O stretching), 1534 cm^−1^ (N = N stretching), 3072 cm^−1^ (aromatic C-H stretching); δ_H_ (600 MHz, CDCl_3_) 7.70 (d, *J* = 12.0 Hz, 2H, Ar–*H*), 7.47 (d, *J* = 12.0 Hz, 2H, Ar–*H*), 2.74 (s, 3H, C*H*_3_), 1.70 (s, 3H, C*H*_3_); δ_C_ (150 MHz, CDCl_3_) 194.34 (-*C* = O), 145.38 (H_3_C-*C* = C-), 132.95 (Ar–*C*-N), 132.31 (H_3_C-C = *C*-), 131.97 (Ar–*C*), 126.67 (Ar–*C*), 116.69 (Ar–*C*), 27.91 (H_3_*C*-C = O), 10.15 (*C*H_3_); Anal. Calcd. for C_11_H_10_BrN_3_O: C, 47.16; H, 3.60; Br, 28.52; N, 15.00. Found: C, 47.29; H, 3.59; Br, 28.47; N, 14.97.

#### 1-(1-(4-chlorophenyl)−5-methyl-1H-1,2,3-triazol-4-yl)ethanone (1e) (Kamalraj et al. 2008)

Yellowish solid; mp:118–119 °C; yield: 2.333 g (%98.99); Rf = 0.46 (n-hexane/ethyl acetate, 2:3); υ_max_ (KBr) 1689 cm^−1^ (C = O stretching), 1509 cm^−1^ (N = N stretching), 3044 cm^−1^ (aromatic C-H Stretching); δ_H_ (600 MHz, DMSO_4_-*d*_*6*_) 7.45 (d, *J* = 12.0 Hz, 2H, Ar–*H*), 7.01 (d, *J* = 12.0 Hz, 2H, Ar–*H*), 2.39 (s, 3H, C*H*_3_), 1.67 (s, 3H, C*H*_3_); δ_C_ (150 MHz, DMSO_4_-*d*_*6*_) 194.99 (-*C* = O), 148.15 (H_3_C-*C* = C-), 134.50 (Ar–*C*-N), 133.27 (H_3_C-C = *C*-), 129.71 (Ar–*C*), 128.96 (Ar–*C*), 119.21 (Ar–*C*), 24.10 (H_3_*C*-C = O), 11.13 (*C*H_3_); Anal. Calcd. for C_11_H_10_ClN_3_O: C, 56.06; H, 4.28; Cl, 15.04; N, 17.83. Found: C, 56.17; H, 4.27; Cl, 15.00; N, 17.79.

### Synthesis of 2-amino-4-(1-(4-substituted phenyl)−5-methyl-1,2,3-triazol-4-yl)thiophene-3-carbonitriles (2a–2e)

**Scheme 2:**
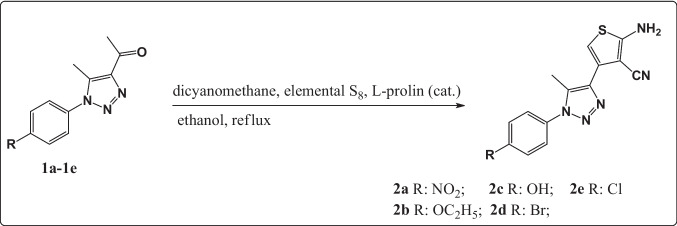
Synthesis of 2-amino-4-(1-(4-substituted phenyl)−5-methyl-1,2,3-triazol-4-yl)thiophene-3-carbonitriles (2a–2e)

In a 100 mL two-necked round-bottom flask, the 1,2,3-triazole derivative (1a–1e, 10 mmol), dicyanomethane (10 mmol), elemental sulfur (S_8_) (10 mmol), catalytic amount of L-proline, and 25 mL of absolute ethanol were combined and stirred. The mixture was refluxed for 3–7 h, and reaction completion was monitored by TLC (Scheme 2). After the reaction, the mixture was cooled to room temperature, poured into an ice–water mixture, and stirred. The resulting solid was filtered, washed with water, dried, and recrystallized from absolute ethanol.

#### 2-amino-4-(5-methyl-1-(4-nitrophenyl)−1H-1,2,3-triazol-4-yl)thiophene-3-carbonitrile (2a)

Yellowish solid; mp:189–190 °C; yield: 2.643 g (%80.99); Rf = 0.39 (n-hexane/ethyl acetate, 2:3); υ_max_ (KBr) 1609 cm^−1^ (N = N stretching), 1353 cm^−1^ (Ar-NO_2_ stretching), 3037 cm^−1^ (aromatic C-H stretching), 3210 cm^−1^ (Ar-NH_2_ stretching),2255 (CN stretching); δ_H_ (600 MHz, CDCl_3_) 8.25 (d, *J* = 12.0 Hz, 2H, Ar–*H*), 8.05 (d, *J* = 12.0 Hz, 2H, Ar–*H*), 7.24 (s, 1H, Ar–H), 6.62 (s, 2H, NH_2_), 2.23 (s, 3H, C*H*_3_); δ_C_ (150 MHz, CDCl_3_) 152.45 (Ar–*C*-NH_2_), 146.13 (Ar–*C*-NO_2_), 144.29 (H_3_C-C = *C*-), 142.00 (H_3_C-*C* = C-), 135.22 (Ar–*C*), 131.39 (Ar–*C*), 126.34 (Ar–*C*), 122.60 (Ar–*C*), 121.60 (Ar–*C*), 113.34 (-*C*N), 80.95 (Ar–*C*-CN), 13.69 (*C*H_3_); Anal. Calcd. for C_14_H_10_N_6_O_2_S: C, 51.53; H, 3.09; N, 25.75; S, 9.83. Found: C, 51.66; H, 3.08; N, 25.72; S, 9.79.

#### 2-amino-4-(1-(4-ethoxyphenyl)−5-methyl-1H-1,2,3-triazol-4-yl)thiophene-3-carbonitrile (2b)

Yellow solid; mp: 167–168 °C; yield: 2.278 g (%70.01); Rf = 0.43 (n-hexane/ethyl acetate, 2:3); υ_max_ (KBr) 1519 cm^−1^ (N = N stretching), 3055 cm^−1^ (aromatic C-H stretching), 3201 cm^−1^ (Ar-NH_2_ stretching), 2269 (CN stretching); δ_H_ (600 MHz, CDCl_3_) 8.05 (d, *J* = 12.0 Hz, 2H, Ar–*H*), 7.24 (s, 1H, Ar–*H*), 6.57–6.56 (m, 4H, Ar–H and NH_2_), 4.32 (q, 2H, *J* = 12.6 Hz, CH_2_), 2.16 (s, 3H, C*H*_3_), 1.55 (t, *J* = 12.6 Hz, 3H, -C*H*_3_); δ_C_ (150 MHz, CDCl_3_) 159.93 (Ar–*C*-OC_2_H_5_), 152.41 (Ar–*C*-NH_2_), 147.04 (H_3_C-C = *C*-), 142.00 (H_3_C-*C* = C-), 138.70 (Ar–*C*), 126.33 (Ar–*C*), 123.98 (Ar–*C*), 122.63 (Ar–*C*), 120.60 (Ar–*C*), 113.34 (-*C*N), 80.54 (Ar–*C*-CN), 63.97 (-*C*H_2_CH_3_), 13.02 (-CH_2_*C*H_3_), 9.46 (*C*H_3_); Anal. Calcd. for C_16_H_15_N_5_OS: C, 59.06; H, 4.65; N, 21.52; S, 9.85. Found: C, 59.20; H, 4.63; N, 21.49; S, 9.80.

#### 2-amino-4-(1-(4-hydroxyphenyl)−5-methyl-1H-1,2,3-triazol-4-yl)thiophene-3-carbonitrile (2c)

Brown solid; mp: 201–202 °C; yield: 2.230 g (%75.00); Rf = 0.47 (n-hexane/ethyl acetate, 2:3); υ_max_ (KBr) 1508 cm^−1^ (N = N stretching), 3057 cm^−1^ (aromatic C-H stretching), 3397 cm^−1^ (O–H stretching), 3200 cm^−1^ (Ar-NH_2_ stretching), 2245 (CN stretching); δ_H_ (600 MHz, CDCl_3_) 7.71 (d, *J* = 12.0 Hz, 2H, Ar–*H*), 7.24 (s, 1H, Ar–*H*), 7.03–6.95 (m, 4H, Ar–H and NH_2_), 5.78 (s, 1H, OH), 2.20 (s, 3H, C*H*_3_); δ_C_ (150 MHz, CDCl_3_) 157.64 (Ar–*C*-OH), 154.31 (Ar–*C*-NH_2_), 149.84 (H_3_C-C = *C*-), 144.91 (H_3_C-*C* = C-), 139.59 (Ar–*C*), 130.48 (Ar–*C*), 126.81 (Ar–*C*), 123.17 (Ar–*C*), 112.48 (-*C*N), 80.38 (Ar–*C*-CN), 9.36 (*C*H_3_); Anal. Calcd. for C_14_H_11_N_5_OS: C, 56.55; H, 3.73; N, 23.55; S, 10.78. Found: C, 56.67; H, 3.72; N, 23.52; S, 10.74.

#### 2-amino-4-(1-(4-bromophenyl)−5-methyl-1H-1,2,3-triazol-4-yl)thiophene-3-carbonitrile (2d)

Yellow solid; mp:180–181 °C; yield: 3.068 g (%85.17); Rf = 0.41 (n-hexane/ethyl acetate, 2:3); υ_max_ (KBr) 1512 cm^−1^ (N = N stretching), 3056 cm^−1^ (aromatic C-H stretching) 3204 cm^−1^ (Ar-NH_2_ stretching), 2250 (CN stretching); δ_H_ (600 MHz, DMSO_4_-*d*_*6*_) 8.00 (d, *J* = 12.0 Hz, 2H, Ar–*H*), 7.68 (d, *J* = 12.0 Hz, 2H, Ar–*H*), 7.31 (s, 1H, Ar–*H*), 6.99 (s, 2H, NH_2_), 2.28 (s, 3H, C*H*_3_); δ_C_ (150 MHz, DMSO_4_-*d*_*6*_) 153.24 (Ar–*C*-NH_2_), 148.38 (H_3_C-C = *C*-), 145.95 (H_3_C-*C* = C-), 138.40 (Ar–*C*), 134.12 (Ar–*C-*Br), 128.21 (Ar–*C*), 125.00 (Ar–*C*), 115.62 (-*C*N), 80.13 (Ar–*C*-CN), 12.20 (*C*H_3_); Anal. Calcd. for C_14_H_10_BrN_5_S: C, 46.68; H, 2.80; Br, 22.18; N, 19.44; S, 8.90. Found: C, 46.82; H, 2.79; Br, 22.14; N, 19.40; S, 8.86.

#### 2-amino-4-(1-(4-chlorophenyl)−5-methyl-1H-1,2,3-triazol-4-yl)thiophene-3-carbonitrile (2e)

Orange solid; mp:183–184 °C; yield: 2.810 g (%88.99); Rf = 0.45 (n-hexane/ethyl acetate, 2:3); υ_max_ (KBr) 1522 cm^−1^ (N = N stretching), 3013 cm^−1^ (aromatic C-H Stretching), 3208 cm^−1^ (Ar-NH_2_ stretching), 2246 (CN stretching); δ_H_ (600 MHz, DMSO_4_-*d*_*6*_) 7.57 (d, *J* = 12.0 Hz, 2H, Ar–*H*), 7.41 (d, *J* = 12.0 Hz, 2H, Ar–*H*), 7.17 (s, 1H, Ar–*H*), 6.93 (s, 2H, NH_2_), 2.28 (s, 3H, C*H*_3_); δ_C_ (150 MHz, DMSO_4_-*d*_*6*_) 154.52 (Ar–*C*-NH_2_), 146.45 (H_3_C-C = *C*-), 144.97 (H_3_C-*C* = C-), 138.00 (Ar–*C*), 135.82 (Ar–*C-*Cl), 132.64 (Ar–*C*-N), 130.50 (Ar–*C*), 129.02 (Ar–*C*), 127.85 (Ar–*C*), 112.51 (-*C*N), 80.43 (Ar–*C*-CN), 11.63 (*C*H_3_); Anal. Calcd. for C_14_H_10_ClN_5_S: C, 53.25; H, 3.19; Cl, 11.23; N, 22.18; S, 10.15. Found: C, 53.39; H, 3.17; Cl, 11.19; N, 22.15; S, 10.12.

### Antibacterial activity

The antibacterial activities of the synthesized 1a-1e and 2a-2e compounds were evaluated against methicillin-susceptible *Staphylococcus aureus* (ATCC 25923) and methicillin-resistant *Staphylococcus aureus* (ATCC 43300) using broth microdilution and agar well diffusion methods.

#### Determination of minimum ınhibitory concentration (MIC) and minimum bactericidal concentration (MBC)

The minimum inhibitory concentrations (MICs) of compounds 1a-1e and 2a-2e were determined by the resazurin microtiter assay (REMA) in 96-well microplates. Two-fold serial dilutions of the compounds were prepared in Mueller–Hinton Broth (MHB) to obtain final concentrations ranging from 0.5 to 256 µg/mL.

Bacterial suspensions prepared from fresh cultures were adjusted to 0.5 McFarland standard and subsequently diluted 1:100. Then, 100 µL of the bacterial suspension was added to each well to achieve a final inoculum of approximately 1 × 10^5^ CFU/mL. Wells containing bacteria without test compounds served as growth controls, whereas wells containing only broth served as sterility controls. Vancomycin was used as the reference antibiotic.

After incubation at 37 °C for 18–24 h, 20 µL of sterile 0.01% (w/v) resazurin solution was added to each well, followed by an additional 3 h incubation under the same conditions. Wells were visually examined for color change. A blue-to-pink or purple color change indicated bacterial growth, whereas wells remaining blue were considered growth-inhibited. The lowest concentration of compound preventing color change was recorded as the MIC.

For determination of the minimum bactericidal concentration (MBC), aliquots from wells showing no visible growth were plated onto Mueller–Hinton Agar (MHA) plates and incubated at 37 °C for 24 h. The lowest concentration that resulted in no bacterial colony formation was defined as the MBC.

Among the tested strains, the MRSA isolate exhibiting the highest susceptibility to the tested compounds was selected for subsequent antibiofilm activity and antibacterial mechanism studies.

### Antioxidant activity

#### DPPH radical scavenging assay

The antioxidant activity of the 1a-1e and 2a-2e compounds was evaluated using the DPPH (2,2-diphenyl-1-picrylhydrazyl) radical scavenging assay following Blois (1958) with slight modifications (Blois 1958). A 0.10 mM DPPH solution was freshly prepared in methanol and protected from light. Sample dilutions (0.005–0.04 mg/mL) were mixed with DPPH (0.5 mL DPPH + 1.5 mL sample) in test tubes, vortexed, and incubated in the dark at room temperature for 30 min. Absorbance was measured at 517 nm using a Shimadzu UV-1800 spectrophotometer, with methanol as blank. DPPH solution without sample served as control; BHT (0.01–0.04 mg/mL) was used as reference.

Scavenging activity (%) was calculated as:

$$Scavenging\, activity\,\left(\%\right)=\frac{{A}_{c}-{A}_{s}}{{A}_{c}}\times 100$$

where Ac and As are the absorbances of the control and test sample, respectively. All experiments were performed in triplicate, and results were expressed as IC_50_ values (μg/mL).

#### ABTS radical scavenging assay

ABTS radical cation scavenging was performed according to Amangeldinova et al. with minor modifications (Amangeldinova et al. 2025). 7 mM ABTS stock solution was reacted with 2.45 mM potassium persulfate and incubated in the dark for 12–16 h. Sample dilutions (0.005–0.04 mg/mL) were mixed with ABTS⁺ working solution (0.5 mL sample + 2.5 mL ABTS⁺), vortexed, and incubated in the dark for 30 min. Absorbance was measured at 734 nm. ABTS⁺ solution without sample served as control; BHT (0.01–0.04 mg/mL) was used as reference.

Scavenging activity (%) was calculated using the same equation as DPPH. All measurements were performed in triplicate, and results were expressed as IC_50_ values (μg/mL).

### Acetylcholinesterase (AChE) ınhibition assay

AChE inhibitory activity of 1a-1e and 2a-2e compounds was determined according to the Ellman method as described in our previous studies (Yıldırım et al. 2025). The assay quantifies the formation of the yellow 5-thio-2-nitrobenzoate anion, produced by the reaction of 5,5′-dithiobis(2-nitrobenzoic acid) (DTNB) with thiocholine released from acetylthiocholine iodide (AChI) hydrolysis.

Stock solutions of hNFs were prepared in methanol and diluted to final concentrations of 0.005–0.04 mg/mL. The reaction mixture contained 50 μL of AChE solution (0.28 U/mL, Electrophorus electricus, Sigma-Aldrich), 100 μL of 1 mM DTNB, 50 μL of sample, and 2.8 mL of 50 mM Tris–HCl buffer (pH 8.0). The mixture was preincubated at 25 °C for 15 min, and the reaction was initiated by adding 50 μL of 15 mM AChI.

The increase in absorbance was monitored at 412 nm using a Shimadzu UV-1800 spectrophotometer for 5 min at room temperature. A control without sample represented 100% enzyme activity, while tacrine served as the positive reference inhibitor. All experiments were performed in triplicate, and results were expressed as mean values of three independent assays.

### Molecular docking study

Molecular docking studies were performed using Maestro 13.8 (Schrödinger Suite 2023–3) following our previously reported protocol with minor modifications (Yıldırım et al. 2025; Öztürk et al. 2026). Three-dimensional structures of the target proteins were retrieved from the Protein Data Bank (PDB): 4EY7 (acetylcholinesterase, Torpedo californica).

The synthesized triazole-thiophene hybrids were drawn and energy-minimized using ChemDraw and LigPrep with the OPLS-4 force field. Docking simulations were conducted using the Glide XP (extra-precision) algorithm. Binding affinities are reported in kcal/mol, where more negative values indicate stronger and more stable ligand–protein interactions. Receptor grids were centered on a representative atom of the co-crystallized ligand, and a default grid box of 20 × 20 × 20 Å was applied for all proteins to ensure uniform sampling of the binding site.

Protein–ligand interactions, including hydrogen bonds, π–π stacking, and hydrophobic contacts, were analyzed using Maestro’s Protein–Ligand Interaction Profiler and Discovery Studio Visualizer (Kumari and Kumar 2025).

### Statistical analysis

All experiments were performed in triplicate, and data are presented as mean ± SD. Statistical comparisons were conducted using GraphPad Prism 9 (GraphPad Software, San Diego, CA, USA). Differences among groups were evaluated by one-way ANOVA followed by Tukey’s post hoc test. Statistical significance was defined as p < 0.05. Distinct letters (a–e) in tables indicate statistically different groups.

## Results and discussion

### Chemistry

For the synthesis of 1,2,3-triazole–thiophene hybrid compounds, 1-(p-substituted phenyl)−4-acetyl-5-methyl-1,2,3-triazole derivatives (1a–1e) were first prepared. The reaction was carried out in a basic medium through a four-step procedure. In the first step, the reaction medium was basified using a sodium carbonate solution. In the second step, azolation was performed at low temperature. In the third step, p-substituted aryl azide derivatives were generated using sodium azide (NaN_3_). In the final step, the 1,2,3-triazole derivatives (1a–1e) were synthesized via a Knoevenagel-type reaction between acetylacetone and the p-substituted aryl azides, catalyzed by potassium carbonate.

The reaction proceeded rapidly and with high yields in the presence of electron-withdrawing substituents (-NO_2_, -Cl, or -Br) (Table 1, Entry 1,4 and 5). In contrast, reactions involving electron-donating groups (-OH or –OC_2_H_5_) required longer reaction times and resulted in lower yields (Table 1, Entry 2 and 3). The reaction times and corresponding yields are summarized in Table 1.

**Table 1:** Reaction times and yields of 1-(p-substituted phenyl)−4-acetyl-5-methyl-1,2,3-triazole derivatives (1a–1e)

Entry	Product	Reaction time (h)	Yield (%)
1	1a	1	99.02
2	1b	4	53.00
3	1c	2	73.98
4	1d	1	98.99
5	1e	1	98.99

The conversion of 1,2,3-triazole derivatives into thiophene-based hybrids was accomplished through a one-pot multicomponent reaction. In this process, the triazole derivatives were treated with dicyanomethane and elemental sulfur in ethanol, using L-proline as a catalyst, leading to the formation of the desired hybrid compounds. The reaction times and corresponding yields are summarized in Table 2.

**Table 2:** Reaction times and yields of the hybrid compounds (2a-2e)

Entry	Product	Reaction time (h)	Yield (%)
1	2a	3	80.99
2	2b	7	70.01
3	2c	5	75.00
4	2d	2	85.17
5	2e	1	88.99

The experimental results indicate that the reaction is influenced by electronic effects. In the aniline ring, higher reactivity and higher yield values were obtained in the presence of electron-withdrawing groups compared to electron-donating groups. An examination of Table 2 reveals that the product yields and reaction times decrease in the order Br > Cl > NO₂ for electron-withdrawing substituents (Table 2, Entry 1, 4 and 5). For electron-donating groups, both the reaction times and product yields are generally lower compared to those with electron-withdrawing substituents. Additionally, the presence of an OH group led to better results than the OC_2_H_5_ group (Table 2, Entry 2 and 3).

### Spectroscopic characterization of the synthesized compounds (1a–1e and 2a–2e)

The structures of the synthesized 1,2,3-triazole derivatives (1a-1e) and the corresponding thiophene carbonitrile derivatives (2a-2e) were successfully confirmed by FT-IR, ^1^H NMR, and ^13^C NMR spectroscopic analyses. In the first series (1a-1e), the characteristic C = O stretching vibrations observed in the FT-IR spectra at ~ 1680–1693 cm^−1^ clearly indicate the presence of the acetyl group, while the bands around ~ 1500–1600 cm^−1^ are consistent with the N = N stretching vibrations of the triazole ring. In the nitro-substituted derivative (1a), the strong absorption at 1349 cm^−1^ confirms the presence of the Ar-NO_2_ group, whereas in the hydroxy derivative (1c), the broad band at 3354 cm^−1^ supports the presence of the phenolic –OH group. In the ^1^H NMR spectra, the aromatic protons appear as two doublets (J ≈ 9.6–12.0 Hz), characteristic of para-disubstituted phenyl rings, confirming the structural symmetry. The singlet signals observed at ~ 2.2–2.7 ppm and ~ 1.6–1.7 ppm are attributed to the acetyl and triazole methyl groups, respectively. In the ^13^C NMR spectra, the signals in the range of ~ 189–197 ppm correspond to the carbonyl carbon, while other aromatic and heterocyclic carbons are observed in their expected regions.

In the second series (2a-2e), the disappearance of the carbonyl band in the FT-IR spectra and the appearance of characteristic C≡N stretching vibrations at ~ 2245–2269 cm^−1^ clearly indicate the formation of the thiophene-3-carbonitrile structure after cyclization. Additionally, the bands observed around ~ 3200–3210 cm^−1^ confirm the presence of the –NH_2_ group, while the peak at ~ 3397 cm^−1^ in the hydroxy derivative (2c) supports the presence of the phenolic –OH group. In the ^1^H NMR spectra, the singlet signals observed around ~ 6.5–7.0 ppm are assigned to amino protons, and the thiophene proton appears as a singlet at ~ 7.1–7.3 ppm, consistent with the proposed structure. Aromatic protons again appear as doublets, in agreement with the para-disubstituted phenyl system. In the ^13^C NMR spectra, the characteristic signals of the nitrile carbon are observed around ~ 112–115 ppm, and the quaternary carbon attached to the nitrile group in the thiophene ring appears clearly around ~ 80 ppm. Furthermore, the elemental analysis results for all compounds are in good agreement with the calculated values, strongly supporting the proposed structures.

### Structure–activity relationship (SAR) of 2-amino-4-(1-(4-substituted phenyl)−5-methyl-1,2,3-triazol-4-yl)thiophene-3-carbonitrile Derivatives (2a-2e)

The structural analysis of the synthesized 1,2,3-triazole–thiophene hybrid compounds revealed that the molecules consist of a thiophene core bearing an amino group at the C-2 position and a nitrile group at the C-3 position, while the C-4 position is linked to a 1,2,3-triazole ring. The triazole ring is further substituted with a para-substituted phenyl group at the N-1 position and a methyl group at the C-5 position. This molecular architecture combines heteroaromatic systems, hydrogen bond-forming functional groups, and lipophilic aromatic substituents, thereby providing a favorable pharmacophoric framework for biological activity.

The thiophene ring, due to its rigid heteroaromatic nature, may facilitate π–π stacking interactions and hydrophobic contacts with the target biomolecules. In addition, the 3-carbonitrile group, acting as a strong electron-withdrawing substituent, may enhance the conjugation of the system and contribute to target binding through dipole–dipole interactions and hydrogen bond-acceptor interactions. The amino group located at the C-2 position of the thiophene ring is considered an important pharmacophoric element capable of functioning as both a hydrogen bond donor and acceptor, which may strengthen ligand–target interactions. Furthermore, this functional group may improve the aqueous solubility of the compounds and modulate the electronic properties of the thiophene ring.

The 1,2,3-triazole ring represents another key structural feature of the synthesized molecules. This heterocyclic moiety is widely recognized as a bioisosteric analogue of the amide functional group and, owing to its high dipole moment and multiple nitrogen atoms, can participate in various non-covalent interactions, including hydrogen bonding and dipole–dipole interactions. Moreover, the triazole ring may act as a molecular linker, contributing to the structural stability and potentially enhancing the metabolic stability of the compounds.

The methyl substituent at the C-5 position of the triazole ring may increase the lipophilicity of the molecules, thereby strengthening interactions with hydrophobic binding pockets and facilitating membrane permeability. Small alkyl substituents at this position are generally considered beneficial for biological activity, whereas bulkier substituents may introduce steric hindrance, potentially reducing the binding affinity toward the biological target.

The structure–activity relationship analysis of the synthesized 1,2,3-triazole–thiophene hybrids (2a-2e) revealed a strong dependence of biological activity on the nature of the para-substituent on the phenyl ring. Antibacterial evaluation demonstrated that compounds bearing electron-withdrawing substituents, particularly halogens, exhibited significantly enhanced potency against both MSSA and MRSA strains, with 2 d and 2e showing the most pronounced activity (MIC up to 2 µg/mL against MRSA). This improvement can be attributed to increased lipophilicity and favorable interactions with bacterial targets. In contrast, acetylcholinesterase (AChE) inhibition followed a different trend, where compounds with a more balanced electronic distribution, notably 2c and 2 d, displayed superior activity (IC_50_ = 25.9–48.5 µg/mL), suggesting that optimal binding within the AChE active site requires a combination of hydrophobic and electronic complementarity. Furthermore, antioxidant activity was more pronounced in compounds with relatively higher electron density (2a and 2c), indicating that radical scavenging ability is governed by electron-donating capacity and resonance stabilization across the triazole–thiophene framework. Overall, the results highlight that electron-withdrawing substituents favor antibacterial potency, whereas balanced or electron-rich systems enhance AChE inhibition and antioxidant activity, underscoring the tunable multifunctional nature of the triazole–thiophene scaffold.

### Antibacterial activity

The nature of the substituents on the phenyl ring appears to play a crucial role in determining the antibacterial activity of the synthesized derivatives. Examination of the MIC values indicated that halogen-substituted derivatives at the para position, particularly the 4-Cl and 4-Br substituted hybrids, exhibited enhanced antibacterial activity against both MSSA and MRSA strains (Table 3). These compounds showed comparatively lower MIC values, suggesting stronger antibacterial potency within the series. The improved activity of these derivatives may be attributed to the increased lipophilicity, modulation of the electronic properties of the phenyl ring, and the potential formation of favorable hydrophobic and halogen-bonding interactions with the biological target.

**Table 3:** Minimum inhibitory concentration (MIC) and minimum bactericidal concentration (MBC) values (µg/mL) of compounds 1a-1e and 2a-2e against methicillin-susceptible and methicillin-resistant *Staphylococcus aureus*

Compounds	MSSA		MRSA	
	MIC	MBC	MIC	MBC
1a	512	1024	512	1024
1b	64	128	64	256
1c	512	1024	512	1024
1d	512	1024	512	1024
1e	512	1024	512	1024
2a	128	512	16	32
2b	128	256	2	8
2c	32	128	2	4
2d	8	16	2	4
2e	8	16	2	4
Vancomycin	1	2	2	4

In contrast, derivatives bearing electron-donating substituents on the phenyl ring (such as –OC_2_H_5_ or –OH) generally displayed moderate to lower antibacterial activity, as reflected by higher MIC values (Table 3). The presence of highly polar substituents, particularly the hydroxyl group, may decrease the overall lipophilicity of the molecule and consequently reduce membrane permeability, which could negatively affect antibacterial activity. However, such substituents may enhance the aqueous solubility of the compounds.

Overall, the obtained results suggest that para-halogen substitution on the phenyl ring represents a favorable structural modification for improving the antibacterial activity of this 1,2,3-triazole–thiophene hybrid scaffold. These findings indicate that lipophilic and electron-withdrawing substituents at the para position may enhance the interaction of the compounds with their biological targets, thereby leading to improved antibacterial efficacy.

In their 2023 study, Szepe et al. synthesized aurone-derived 1,2,3-triazole compounds and investigated their antibacterial effects against MSSA and MRSA (Szepe et al. 2023). According to the findings, all four synthesized compounds exhibited inhibition against MSSA at relatively low IC_50_ concentrations; however, only the compounds coded AT125 and AT137 were also able to inhibit MRSA at concentrations below 6 μM. These compounds were reported to carry cyano and bromo substituents on the aromatic ring attached to the triazole moiety.

In another study conducted by Negi et al., 35 metronidazole–triazole hybrid compounds were synthesized and their in vitro anti-MRSA activities were evaluated (Negi et al. 2016). The results indicated that compounds bearing halogen substituents on the benzene ring attached to the triazole moiety exhibited the highest antibacterial activity.

### Acetylcholinesterase (AChE) inhibition

**Scheme 3:**
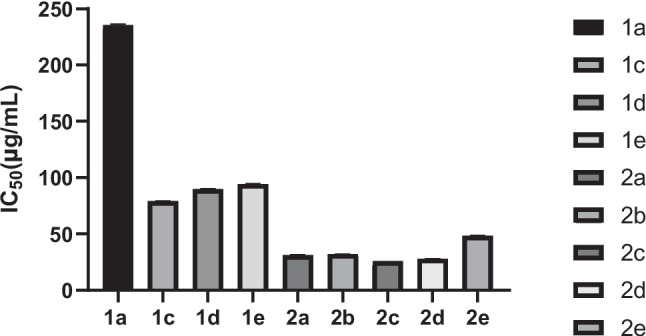
IC_50_ values (µg/mL) of the tested compounds against AChE enzyme

The inhibitory effects of the synthesized compounds on AChE were evaluated based on their IC_50_ values (Scheme 3). Lower IC_50_ values indicate stronger inhibition, as the compound can suppress 50% of enzymatic activity at lower concentrations. Significant differences in AChE inhibitory activity were observed among the tested series. IC_50_ values (µg/mL), regression equations, and R^2^ coefficients for AChE inhibitory activity of the tested compounds are summarized in Table 4.

**Table 4:** IC_50_ values (µg/mL), regression equations, and R^2^ coefficients for AChE inhibitory activity of the tested compounds

	IC_50_ (µg/mL)	R^2^	Denklem
1a	236.079 ± 0.763^a^	0.9953	y = 0.2052x + 1.5566
1c	78.928 ± 0.051^d^	0.9931	y = 0.6145x + 1.4989
1d	89.501 ± 0.353^c^	0.995	y = 0.57x—1.0178
1e	94.391 ± 0.277^b^	0.9954	y = 0.5462x—1.5566
2a	31.454 ± 0.321^f^	0.9844	y = 1.4726x + 3.6804
2b	31.808 ± 0.136^f^	0.9919	y = 1.4848x + 2.7717
2c	25.914 ± 0.061^ h^	0.9869	y = 1.787x + 3.6913
2d	27.664 ± 0.238^ g^	0.9787	y = 1.698x + 3.0271
2e	48.490 ± 0.347^e^	0.9914	y = 1.3392x + 1.9266

Hybrid compounds exhibited IC_50_ values ranging from 25.914 to 48.490 µg/mL, indicating higher potency relative to the 1a-1e series. Notably, 2c (25.914 ± 0.061 µg/mL) and 2 d (27.664 ± 0.238 µg/mL) were the most potent inhibitors. Compounds 2a (31.454 ± 0.321 µg/mL) and 2b (31.808 ± 0.136 µg/mL) showed comparable activity, while 2e (48.490 ± 0.347 µg/mL) displayed moderate inhibition. In contrast, the 1a-1e series showed higher IC_50_ values (78.928–236.079 µg/mL), with 1a (236.079 ± 0.763 µg/mL) being the weakest inhibitor. Compounds 1c, 1 d, and 1e exhibited intermediate activity but remained less effective than the hybrid compounds. When the acetylcholinesterase inhibitory effects of triazole-hybrid compounds in the literature are examined, a study by Li et al. reported the synthesis of novel 1,2,3-triazole derivatives and the determination of their enzyme inhibition values (Li et al. 2016). It was observed that an increase in the number of halogen substituents on the ring led to lower IC_50_ values.

In another study conducted in 2015 by Mohammadi-Khanaposhtani et al., 1,2,3-triazole–acridone hybrid compounds were synthesized and their acetylcholinesterase inhibitory activities were investigated (Mohammadi-khanaposhtani et al. 2015). It was determined that the hybrid compound containing a chloro substituent at the para position of the aromatic ring attached to the triazole moiety exhibited the best activity (IC_50_ = 7.31 μM).

The regression analysis of dose–response curves yielded R^2^ values between 0.9787 and 0.9954, confirming good model fit and reliable IC_50_ determination. Higher slopes in the hybrid compounds indicate a more pronounced inhibition response with increasing concentration, reflecting a sharper dose–response relationship.

Evaluation of percent inhibition at discrete concentrations further corroborated these findings. The hybrid compounds produced significant inhibition even at low concentrations (15 µg/mL), with 2a, 2b, 2c, 2 d, and 2e achieving approximately 31.38%, 27.71%, 32.93%, 28.54%, and 25.80% inhibition, respectively. At 30 µg/mL, inhibition increased markedly (e.g., 2c, 62.27%; 2 d, 61.46%), and at 50 µg/mL, 2c (89.33%) and 2 d (83.41%) displayed the highest inhibition, indicating strong dose-dependent effects.

By contrast, compounds in the 1a-1e series required higher concentrations to achieve comparable inhibition. At 30 µg/mL, 1a, 1c, 1 d, and 1e showed only 7.11%, 23%, 16%, and 12.67% inhibition, respectively. Even at 100 µg/mL, inhibition remained lower than that of hybrid compounds, and 1a exhibited only 52.67% inhibition at 250 µg/mL, confirming its weak potency.

Overall, the hybrid compounds demonstrated earlier and stronger AChE inhibition, with 2c and 2 d emerging as the most potent candidates. These results suggest that structural features within the hybrids enhance interactions with the AChE active site, contributing to their superior inhibitory activity. ADME studies were performed for all synthesized compounds, and it was determined that compounds 1a-1e cross the blood–brain barrier (BBB), whereas compounds 2a-2e do not. In addition, the bioavailability radar analysis and BOILED-Egg ADME diagram for compounds 1a-1e and 2a-2e, as well as the WLOGP and TPSA data, have been included in the Supplementary file.

One-way ANOVA revealed statistically significant differences among the IC_50_ values of the nine tested compounds (α = 0.05). Post hoc analysis using Tukey’s multiple comparison test indicated that the majority of the 36 pairwise comparisons were highly significant, demonstrating pronounced differentiation in inhibitory potency among the compounds. The mean IC_50_ values spanned a wide range (25.96–235.5 µg/mL), with 1a exhibiting the highest IC_50_ (235.5 µg/mL) and significantly weaker inhibition than all other compounds (*p* < 0.0001). Conversely, 2c (25.96 µg/mL) and 2 d (27.83 µg/mL) displayed the lowest IC_50_ values, indicating the strongest inhibitory activity. In the intra-group comparison of the synthesized hybrid compounds, the statistical comparisons for all compounds were calculated as *p* < 0.0001, except for 2a vs 2b (*p* = 0.0234) and 2c vs 2 d (*p* = 0.5992). For the triazole compound group, the comparison between 1 d and 1e was *p* = 0.0222, while the others were calculated as *p* < 0.0001 (Supplementary file).

The Compact Letter Display (CLD) derived from Tukey’s test provided a hierarchical grouping of compounds based on their activity. Groups assigned different letters represent statistically significant differences, whereas compounds sharing the same letter do not differ significantly. 2c alone occupied the highest activity group (h), statistically distinct from all other compounds. 2a and 2b were assigned to the same group (f) and showed no significant difference from each other. 1a fell into the lowest activity group (A), significantly separated from all others.

Overall, the results indicate that the hybrid series exhibits substantially higher AChE inhibitory activity compared to the 1a-1e series. The presence of multiple hybrid compounds in the upper activity groups (h, g, f, e) suggests that structural modifications within this series enhance interactions with the enzyme active site. The exclusive placement of 2c in the top statistical group (h) highlights its potential as an optimized lead compound, while 2 d in the immediately lower group (g) illustrates how minor structural variations can markedly affect potency. The grouping of 2a and 2b in the same category (f) implies similar pharmacophoric properties or comparable binding configurations within the active site. In contrast, the 1a-1e series clustered in lower activity groups (a–d) reflects comparatively weaker enzyme affinity, likely due to structural differences that reduce interaction efficiency.

### Antioxidant activity

The antioxidant potential of the synthesized compounds was evaluated using DPPH and ABTS radical scavenging assays, and the results are presented in Table 5. IC_50_ values exhibited substantial variation across the tested compounds, indicating differential radical scavenging capacity.

**Table 5:** Antioxidant activities of the compounds

Compound	DPPH		ABTS	
	R^2^	IC_50_ (µg/mL)	R^2^	IC_50_ (µg/mL)
1a	-	-	0.9965	34.20
1b	0.9939	11.07	0.9900	4.88
1c	-	-	0.9996	160.30
1d	-	-	0.9483	124.40
1e	0.9790	2470.00	0.9918	135.20
2a	0.9909	5.01	0.9986	94.70
2b	0.9875	532.20	0.9986	49.40
2c	0.9741	105.30	0.9941	16.00
2d	0.9506	77.10	0.9941	72.00
2e	0.9787	100.10	0.9979	65.10
BHT		3.5		3.4

For the DPPH assay, compounds 2a (5.01 µg/mL), 1b (11.07 µg/mL), and 2 d (77.10 µg/mL) showed the most potent activity, while compounds 1e (2470.00 µg/mL) and 2b (532.20 µg/mL) exhibited weaker scavenging effects. In the ABTS assay, 1b (4.88 µg/mL) and 2c (16.00 µg/mL) displayed the strongest radical inhibition, whereas 1c (160.30 µg/mL) and 1e (135.20 µg/mL) were less active.

Overall, the data indicate that 2a and 2c demonstrate significant antioxidant activity in both assays, whereas the 1a-1e compounds generally exhibited lower radical scavenging potential. Variation in IC_50_ values across the series suggests that structural modifications within the hybrid compounds enhance free radical neutralization, highlighting their potential as antioxidant candidates. Shaikh et al. reported that the newly synthesized and characterized 1,2,3-triazole derivatives incorporating coumarin exhibited radical scavenging activity against DPPH, with IC_50_ values ranging from 15.20 to 40.36 µg/mL (Shaikh et al. 2015). It was determined that the activities of the synthesized compounds varied depending on the substituents they contained. Compounds bearing NO_2_ and H substituents demonstrated higher radical scavenging activity.

In another study, the ABTS radical scavenging activities of nitrone-containing 1,2,3-triazole derivatives were evaluated. While some of the synthesized compounds did not exhibit any radical scavenging activity, compound 8f demonstrated 34.3% radical scavenging activity (Hadjipavlou-litina et al. 2023). Within this series, the presence of a fluorine (F) substituent in the structure was found to enhance ABTS radical scavenging activity.

### Molecular docking study

The synthesized compounds exhibited docking scores ranging from − 5.426 to − 6.616 kcal/mol, while compound 2e demonstrated the strongest interaction with a docking score of − 6.844 kcal/mol. As illustrated in Table 6, the aromatic ring of compound 2e formed a π–π stacking interaction with PHE338. In addition, the five-membered heterocyclic ring of the compound established another π–π stacking interaction with TYR341. Furthermore, the nitrogen atom present in the molecular structure formed a salt bridge interaction with ARG296, which likely contributes to the stabilization of the ligand within the binding pocket.

**Table 6:** Molecular docking scores and Glide emodel values of synthesized compounds

Compounds	Protein	Docking score	Glide emodel	MM-GBSA binding energy
1a	4EY7	−6.540	−47.589	−49.80
1b	4EY7	−5.444	−49.985	−56.81
1c	4EY7	−5.426	−48.184	−46.44
1d	4EY7	−5.985	−47.430	−43.31
1e	4EY7	−6.117	−47.224	−55.22
2a	4EY7	−6.327	−63.159	−57.45
2b	4EY7	−5.773	−61.081	−61.23
2c	4EY7	−6.616	−58.069	−61.27
2d	4EY7	−6.625	−62.818	−54.16
2e	4EY7	−6.844	−56.297	−52.61
Tacrine	4EY7	−6.634	−49.372	−51.84

Among the other compounds, compound 2 d, which showed the second highest binding affinity, formed π–π stacking interactions with TYR124 and PHE338, while a salt bridge interaction was observed with GLY121.

**Scheme 4:**
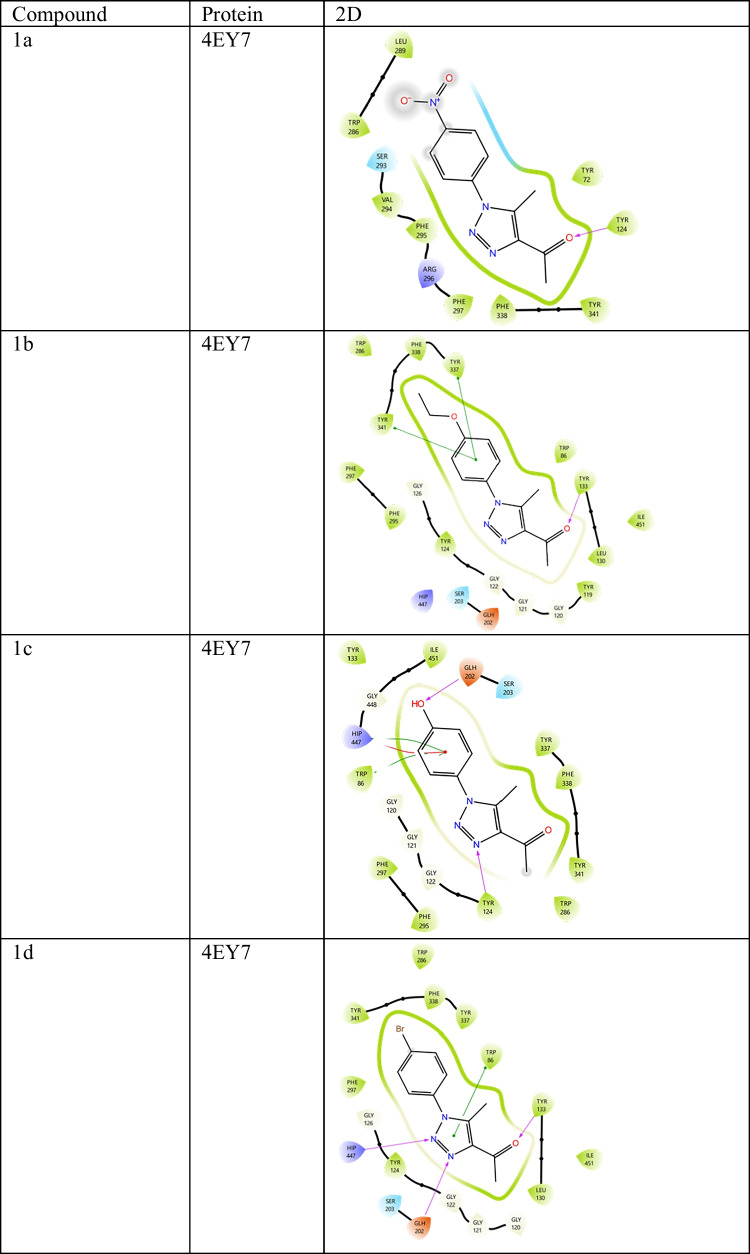

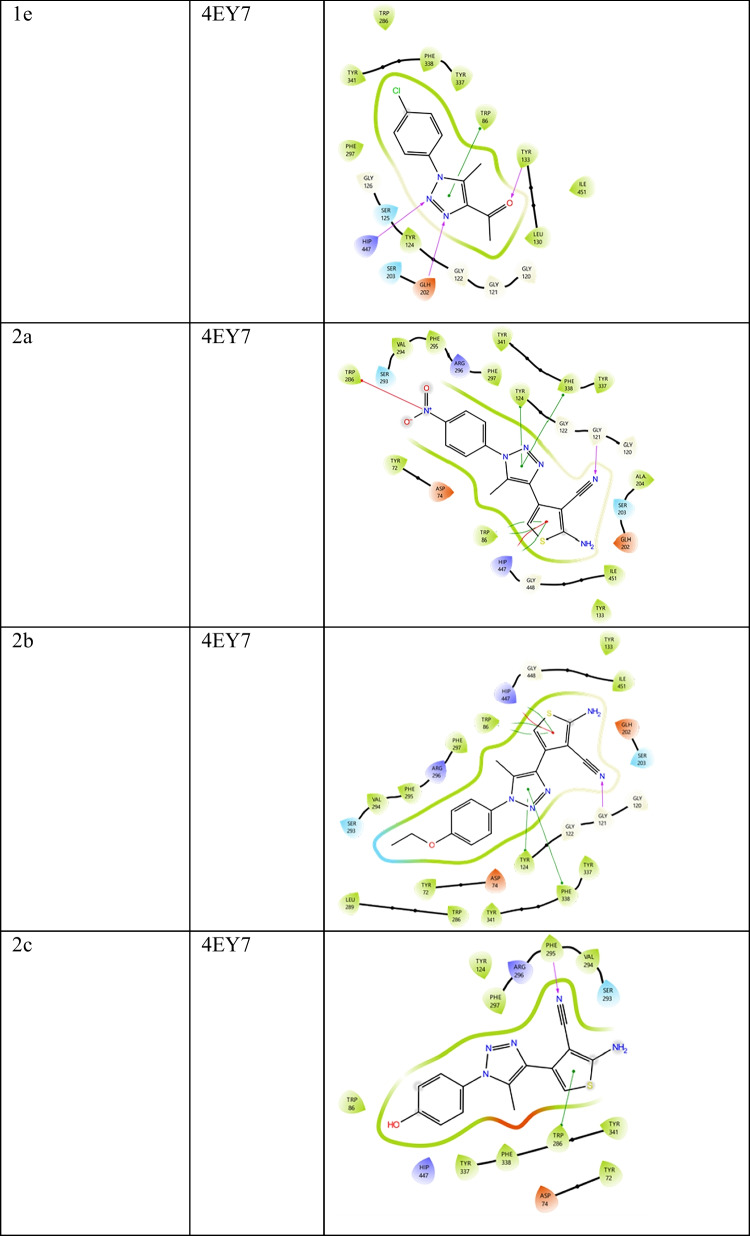

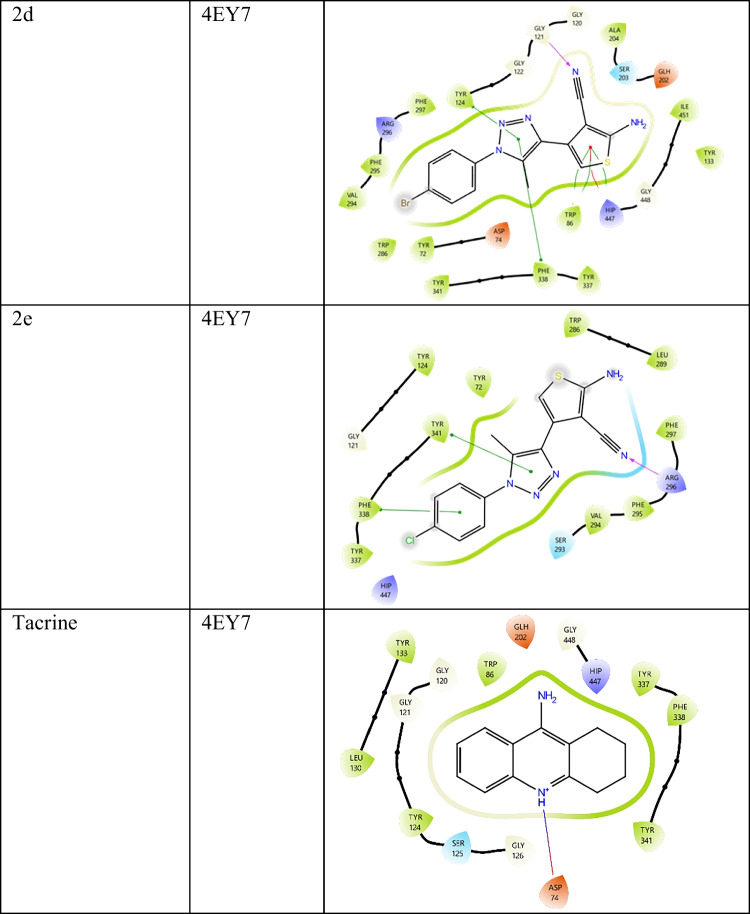
2D binding interactions of compounds in the active site of targeted protein

In the case of compound 2c, a π–π stacking interaction was detected with TRP286, whereas a salt bridge interaction was formed with PHE295, suggesting additional stabilization of the ligand–protein complex. These results suggets that observed biological activities of these compounds are supperted by the strong and spesific ligand–protein interactions at the molecular level (Scheme 4).

## Conclusion

In this study, a new series of 1,2,3-triazole–thiophene hybrid compounds (2a–2e) was successfully designed, synthesized, and comprehensively evaluated for their biological activities. The synthetic strategy, involving the preparation of 1-(p-substituted phenyl)−4-acetyl-5-methyl-1,2,3-triazoles (1a–1e) followed by a one-pot multicomponent reaction with dicyanomethane and elemental sulfur in the presence of L-proline, proved to be efficient, affording the target compounds in good yields. Substituent effects played a significant role, with electron-withdrawing groups promoting higher reaction rates and yields compared to electron-donating groups.

Biological evaluation revealed that the incorporation of the thiophene moiety markedly enhanced activity across all tested assays. The synthesized hybrids exhibited significant antibacterial activity against both methicillin-susceptible and methicillin-resistant *Staphylococcus aureus*, with compounds 2 d and 2e emerging as the most potent candidates (MIC = 2 µg/mL). Structure–activity relationship analysis indicated that para-halogen substituents (Cl and Br) positively influence biological activity, likely due to increased lipophilicity and favorable hydrophobic and halogen-bonding interactions.

In addition, the hybrid compounds demonstrated improved AChE inhibitory activity compared to their precursor analogues, with compounds 2c (IC_50_ = 25.914 ± 0.061 µg/mL) and 2 d (IC_50_ = 27.664 ± 0.238 µg/mL)showing the most pronounced effects, suggesting enhanced binding interactions within the enzyme active site. Antioxidant studies further supported the multifunctional nature of these compounds, with several derivatives exhibiting moderate to strong radical scavenging activity. For the DPPH assay, compounds 2a (5.01 µg/mL), 1b (11.07 µg/mL), and 2 d (77.10 µg/mL) showed the most potent activity, while compounds 1e (2470.00 µg/mL) and 2b (532.20 µg/mL) exhibited weaker scavenging effects. In the ABTS assay, 1b (4.88 µg/mL) and 2c (16.00 µg/mL) displayed the strongest radical inhibition, whereas 1c (160.30 µg/mL) and 1e (135.20 µg/mL) were less active.

Molecular docking studies (PDB ID: 4EY7) were consistent with the experimental findings, revealing favorable binding affinities and key interactions, including π–π stacking and salt-bridge formations with critical amino acid residues in the active site.

Overall, the results clearly demonstrate that 1,2,3-triazole–thiophene hybridization is an effective strategy for the development of multifunctional bioactive molecules. Among the synthesized compounds, 2c–2e stand out as promising lead candidates. This study not only provides valuable insights into structure–activity relationships but also highlights the potential of this scaffold for further optimization toward therapeutic applications, particularly in the treatment of neurodegenerative disorders and antibiotic-resistant infections.

## Supplementary Information

Below is the link to the electronic supplementary material.

Supplementary file1 (DOCX 4145 KB)

## Data Availability

All source data for this work (or generated in this study) are available upon reasonable request.
