# Supplementary material for: Novel 1,2,3-triazole–thiophene hybrids: synthesis, anti-MRSA/MSSA activity, antioxidant potential, and acetylcholinesterase ınhibition supported by molecular docking
Source: Naunyn Schmiedebergs Arch Pharmacol. 2026 May 5;399(10):15955–70. doi: 10.1007/s00210-026-05402-w (PMC13391755; doi:10.1007/s00210-026-05402-w)
Supplement: Supplementary file 1 — Supplementary file1 (DOCX 4145 KB) [file 210_2026_5402_MOESM1_ESM.docx]

**SUPPLEMENTARY MATERIAL**

**FOR**

**Novel 1,2,3-Triazole–Thiophene Hybrids: Synthesis, Anti-MRSA/MSSA Activity, Antioxidant Potential, and Acetylcholinesterase Inhibition Supported by Molecular Docking**

Mehmet Erşatır*

*Department of Chemistry, Faculty of Art and Science, Cukurova University, Adana 01330, Türkiye

Corresponding author e-mail: [mersatir@cu.edu.tr](mailto:mersatir@cu.edu.tr)

,

**Supporting Information**

**Contents:**

| **I** | Instrumentation Details | **S2** |
| --- | --- | --- |
| **II** | ^1^H and ^13^C NMR spectra of compounds **1a-1e** and **2a-2e** | **S3-S13** |
| **III** | MIC results of the compounds **1a-1e** and **2a-2e** | **S14** |
| **IV** | FT-IR spectra of compounds **1a-1e** and **2a-2e** | **S15-S19** |
| **V** | Table S1. Elemental Analysis Results of compounds **1a-1e** and **2a-2e** | **S20** |
| **VI** | Table S2. ADME Pharmacokinetics and Druglikeness properites of compounds | **S20** |
| **VII** | Table S3. Bioavailability radar analysis and The BOILED-Egg ADME diagram of the compounds **1a-1e** and **2a-2e** WLOGP vs. TPSA | **S22** |

**I. Instrumentation Details**

^1^H and ^13^C NMR spectra were recorded in CDCl_3_ or DMSO_4_-*d_6_* [using the solvent peak as internal reference (DMSO_4_-*d_6_*: δH 2.50; δC 39.51; CDCl_3_: δH 7.27; δC 77.00) on a Bruker Avance III HD 600 MHz at Inönü University Scientific and Technological Research Center and operating at 600 MHz and 150 MHz, respectively. All chemical shift values are quoted in ppm and coupling constants quoted in Hz. Multiplicities are indicated, s (singlet), d (doublet), t (triplet), q (quartet), sept (septet), m (multiplet), br s (broad singlet).

The melting points were determined on a Electro thermal 9100 melting-point instrument and are uncorrected. IR spectra were recorded on a Perkin-Elmer 55148 spectrometer. Elemental analyses were measured on a Thermo Flash 2000 Organic Elemental Analyzer.

**II. ^1^H and ^13^C spectra of compounds (1a-1e and 2a-2e)**

^1^H NMR spectrum of compound **1a**


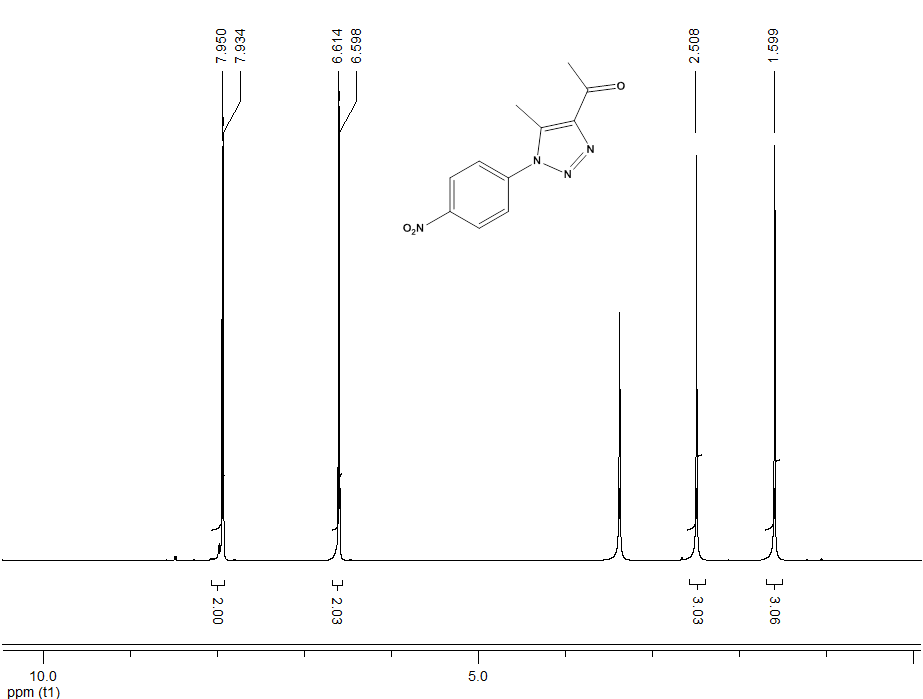


^13^C NMR spectrum of compound **1a**


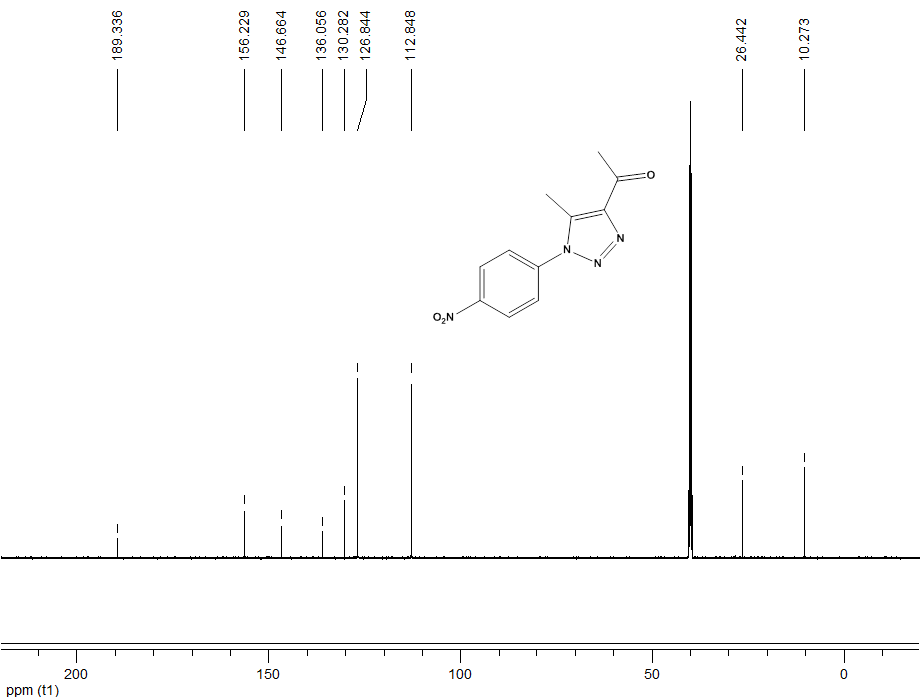


^1^H NMR spectrum of compound **1b**


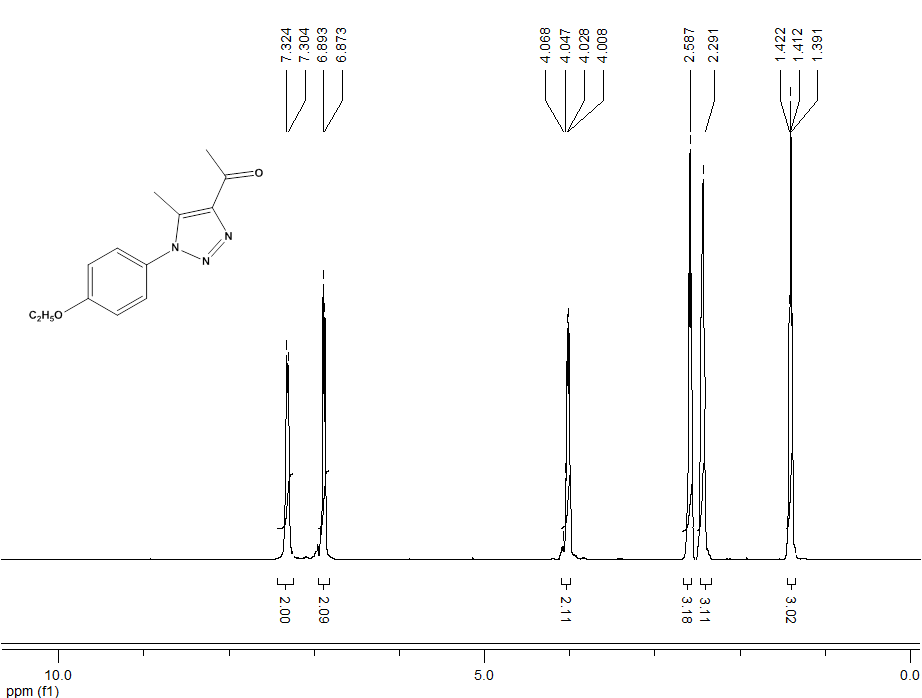


^13^C NMR spectrum of compound **1b**


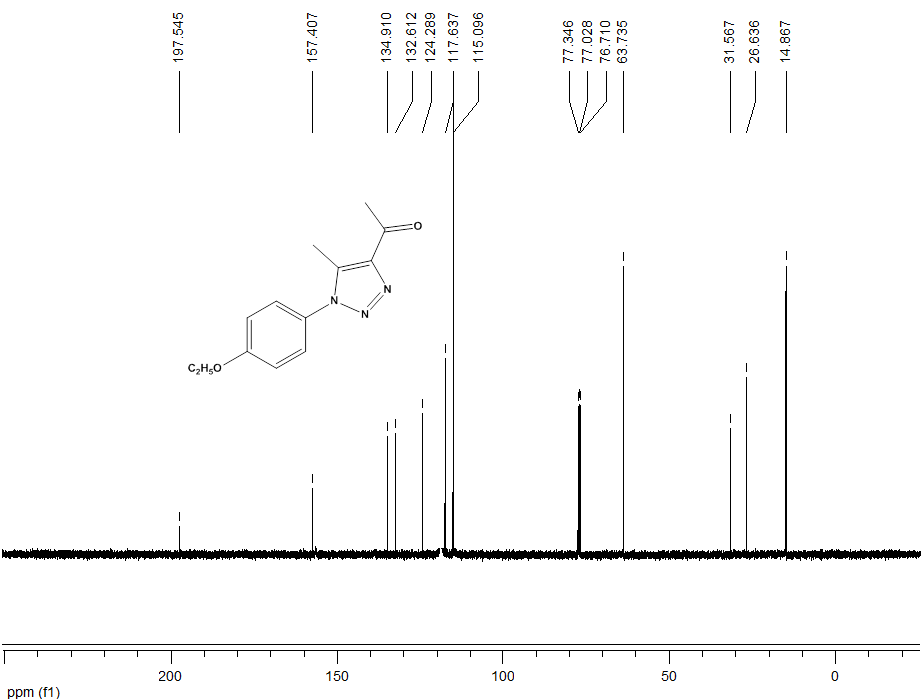


^1^H NMR spectrum of compound **1c**


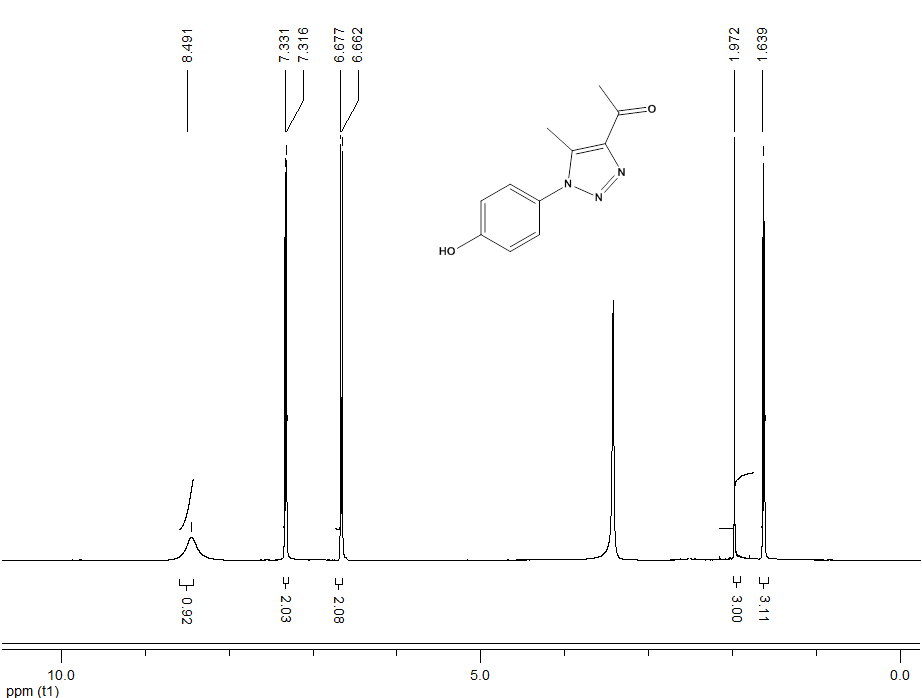


^13^C NMR spectrum of compound **1c**


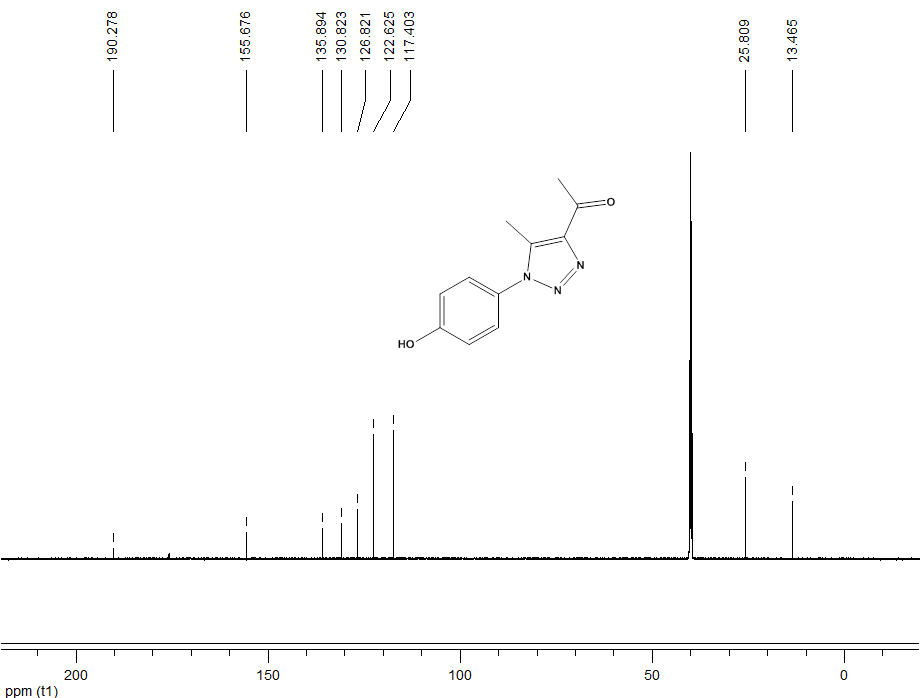


^1^H NMR spectrum of compound **1d**


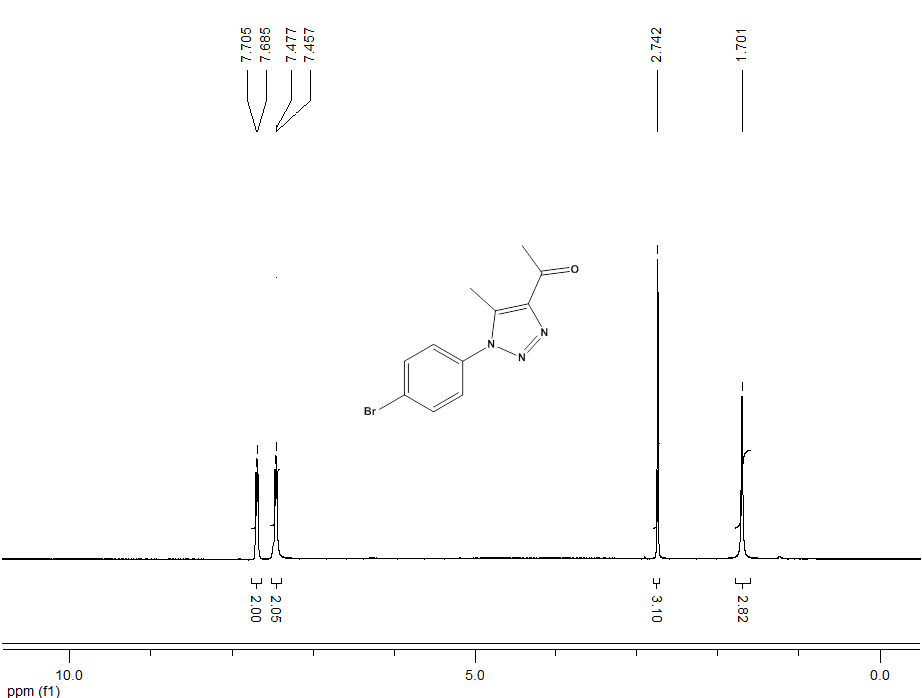


^13^C NMR spectrum of compound **1d**


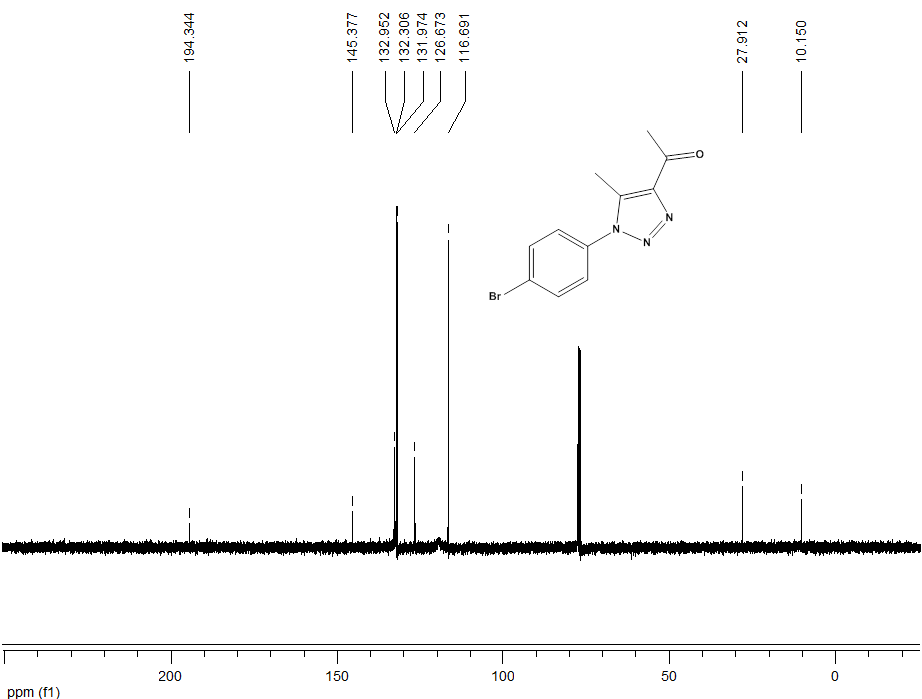


^1^H NMR spectrum of compound **1e**


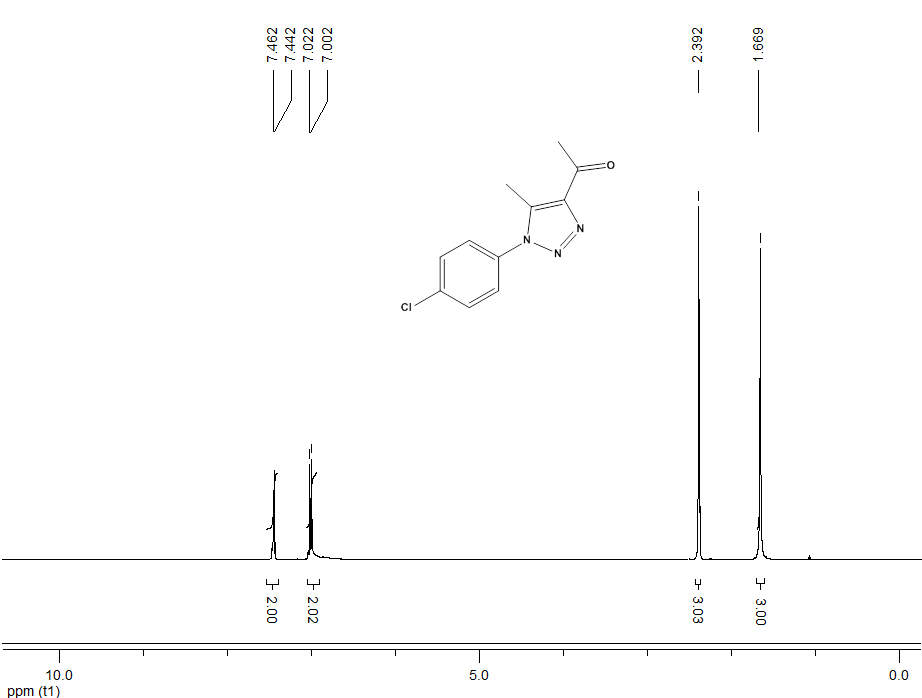


^13^C NMR spectrum of compound **1e**


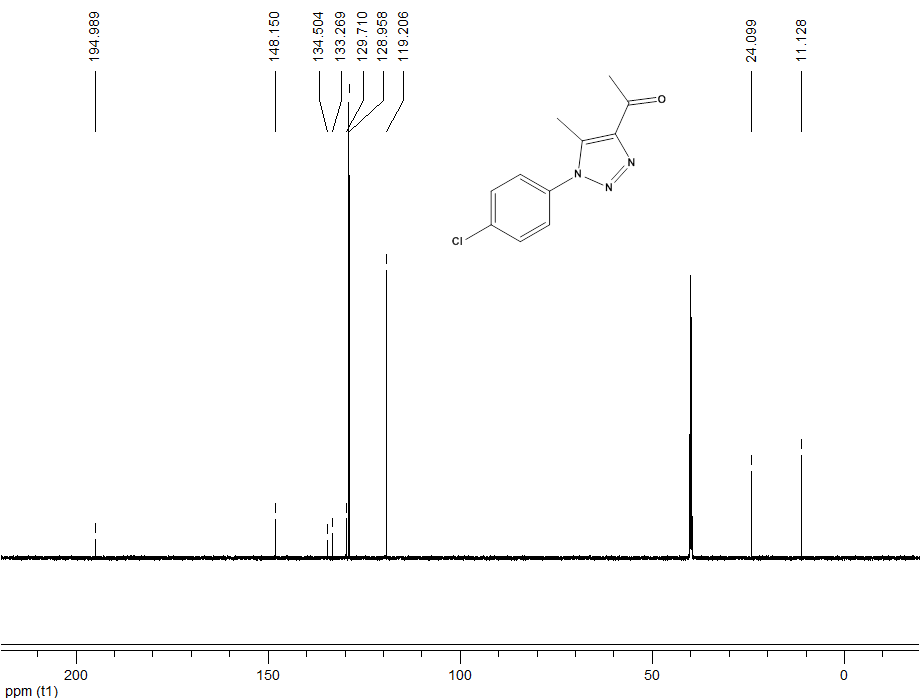


^1^H NMR spectrum of compound **2a**


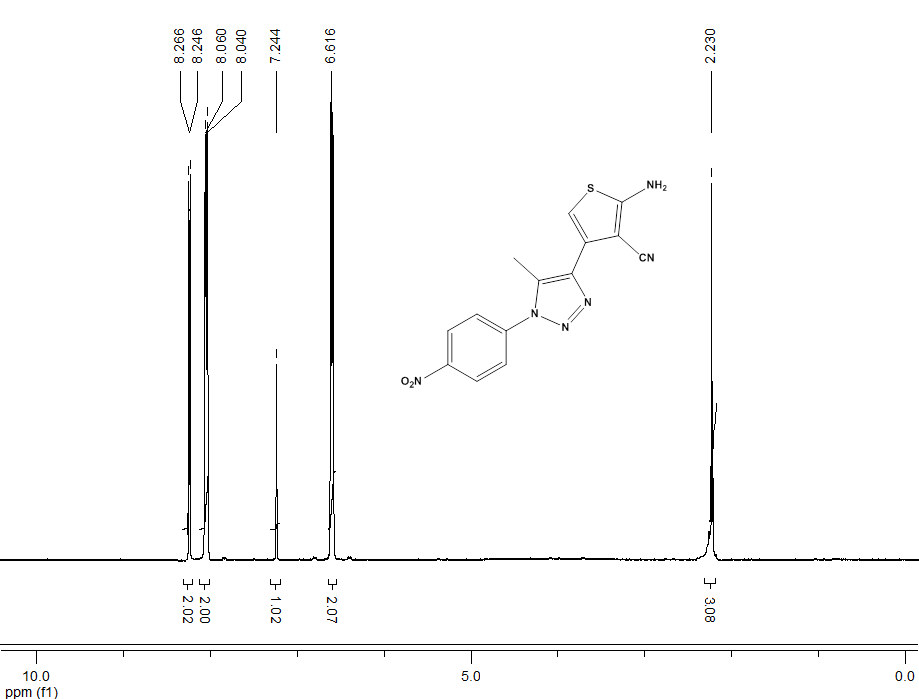


^13^C NMR spectrum of compound **2a**


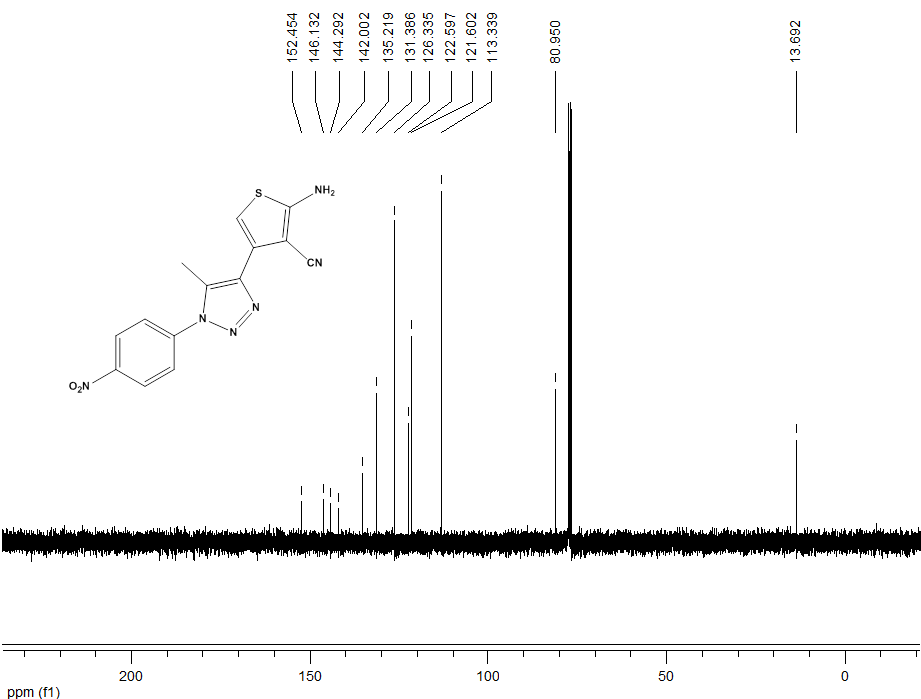


^1^H NMR spectrum of compound **2b**


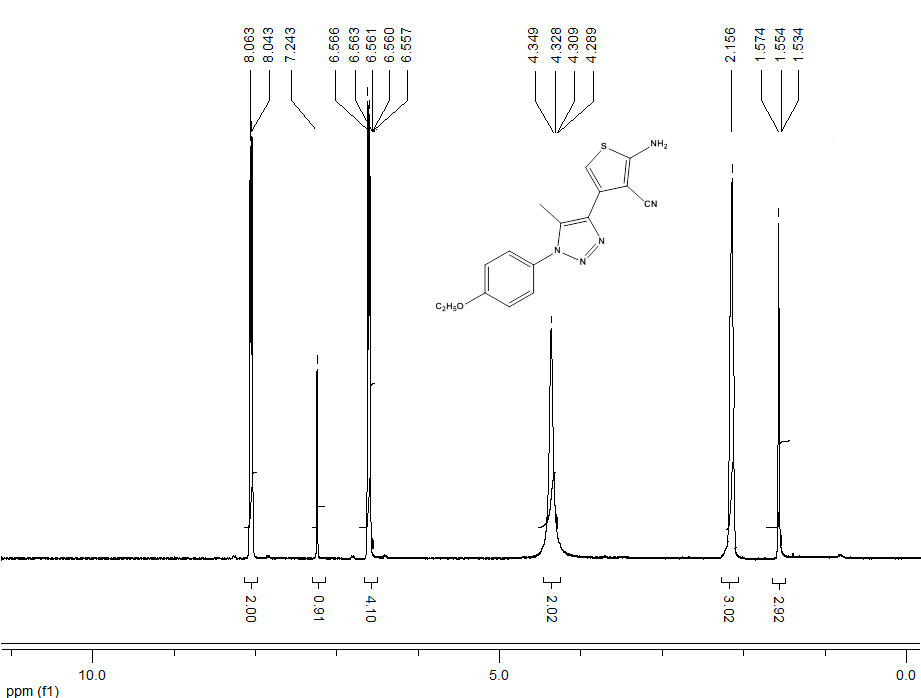


^13^C NMR spectrum of compound **2b**


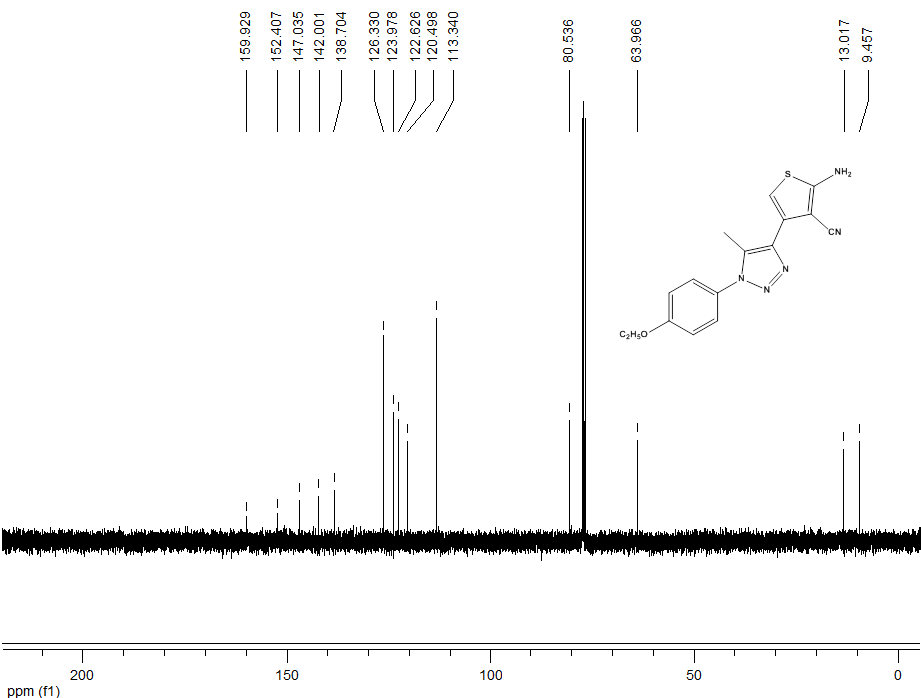


^1^H NMR spectrum of compound **2c**


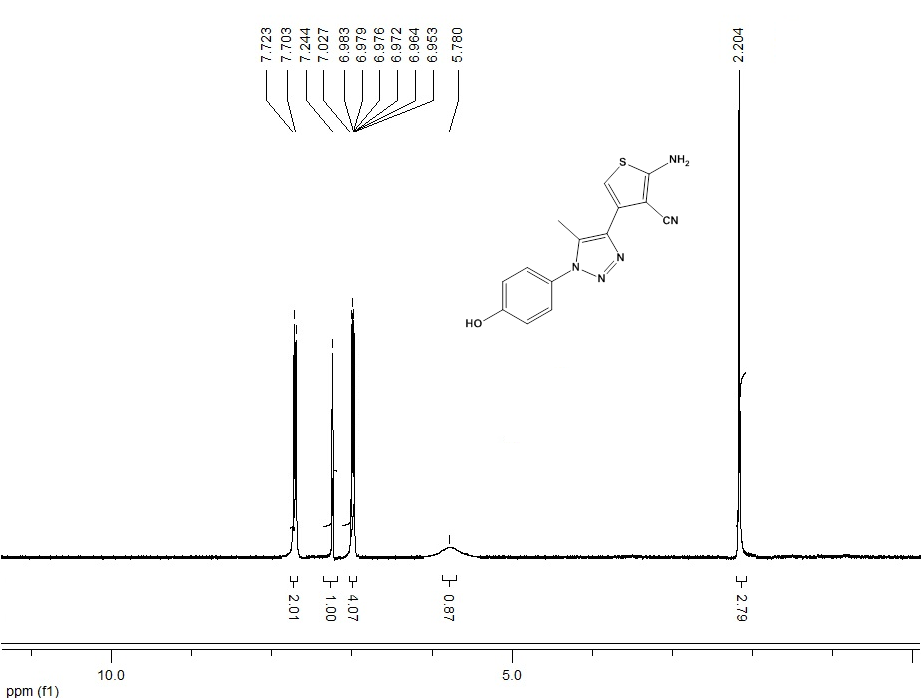


^13^C NMR spectrum of compound **2c**


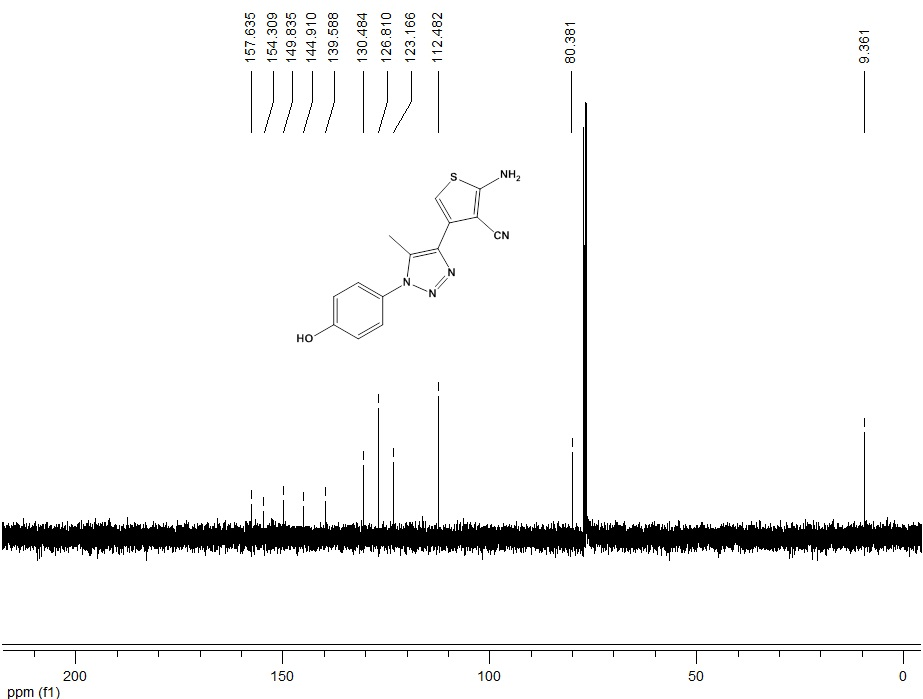


^1^H NMR spectrum of compound **2d**


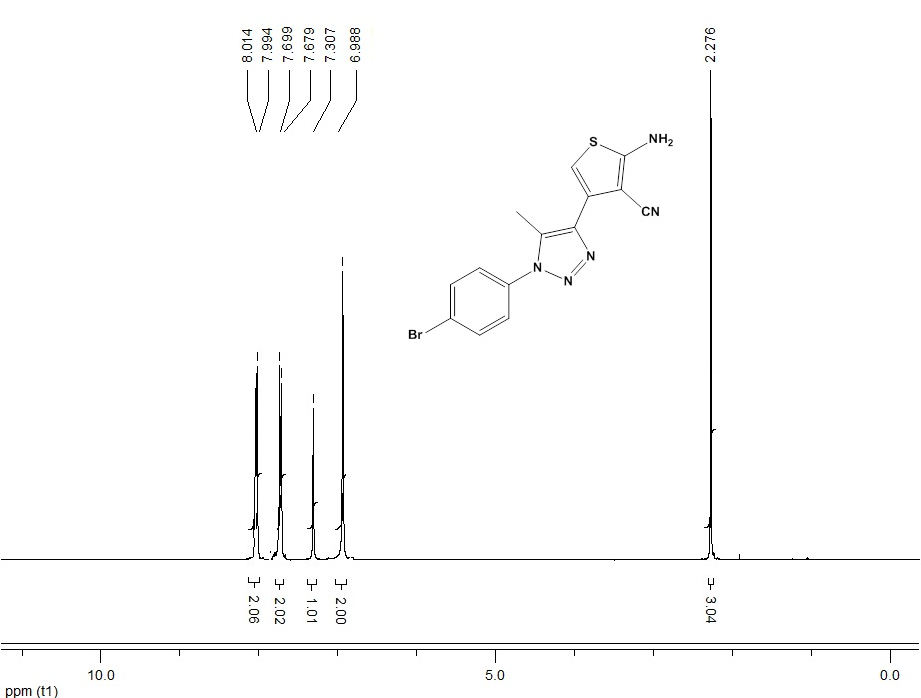


^13^C NMR spectrum of compound **2d**


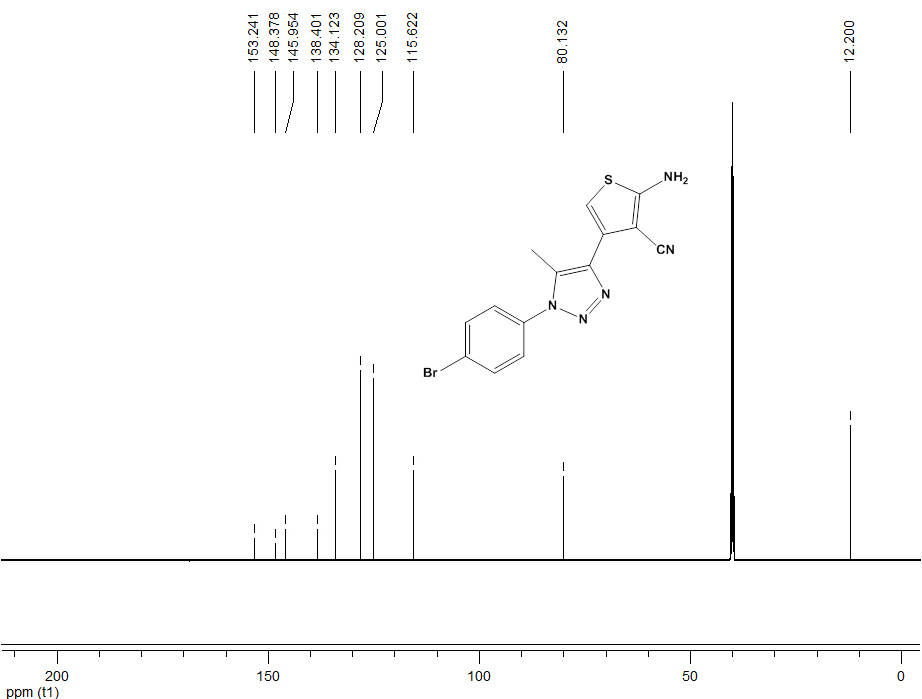


^1^H NMR spectrum of compound **2e**


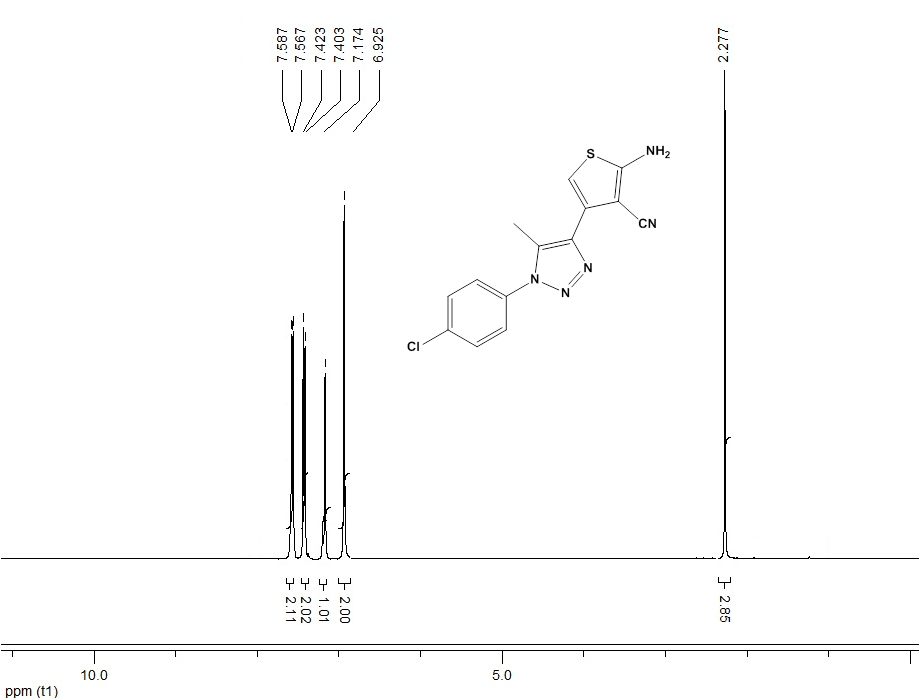


^13^C NMR spectrum of compound **2e**


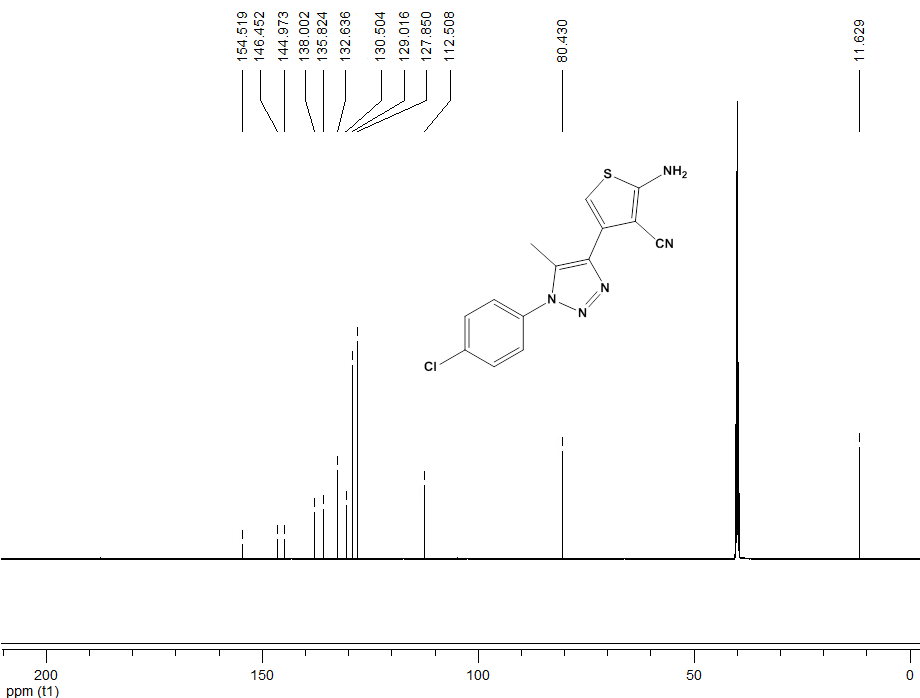


**III. MIC Results of the Compounds**

**
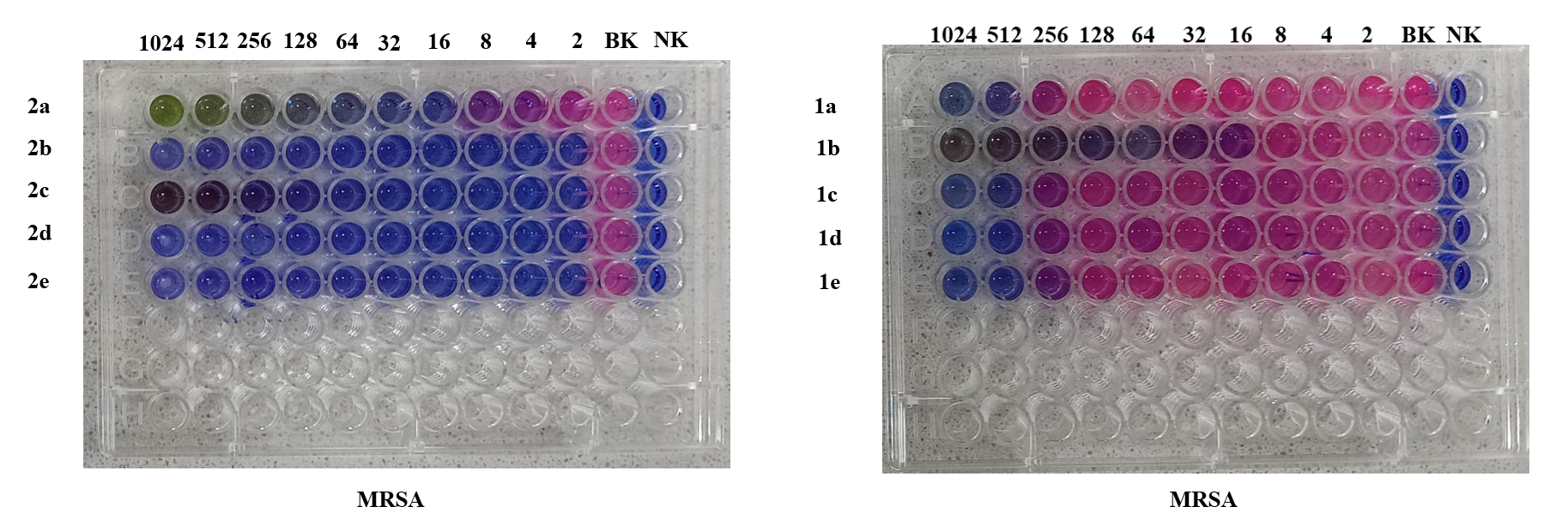
**

**
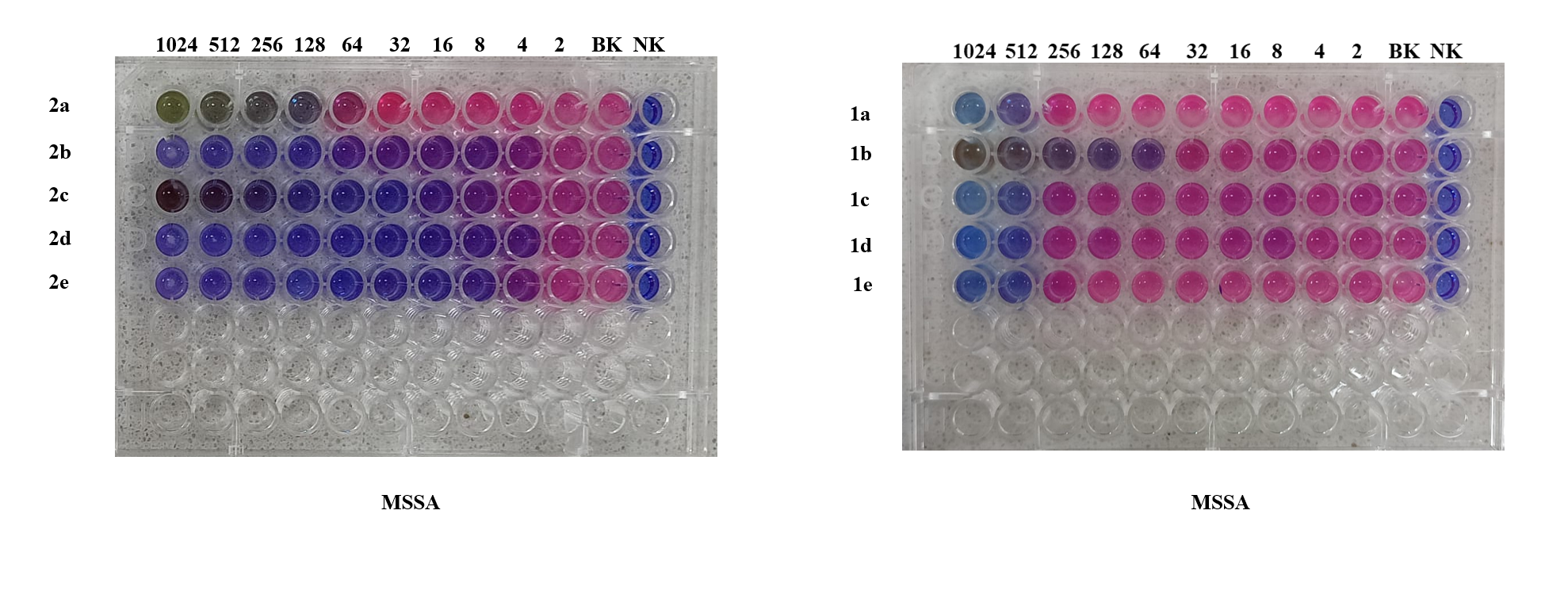
**

**IV. FT-IR spectra of compounds (1a-1e and 2a-2e)**


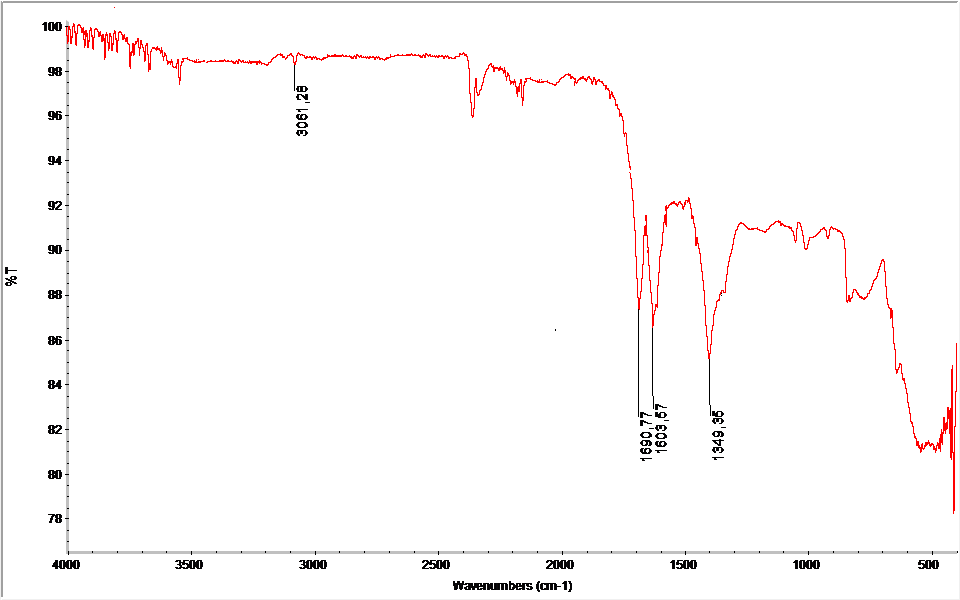


FT-IR spectrum of compound **1a**


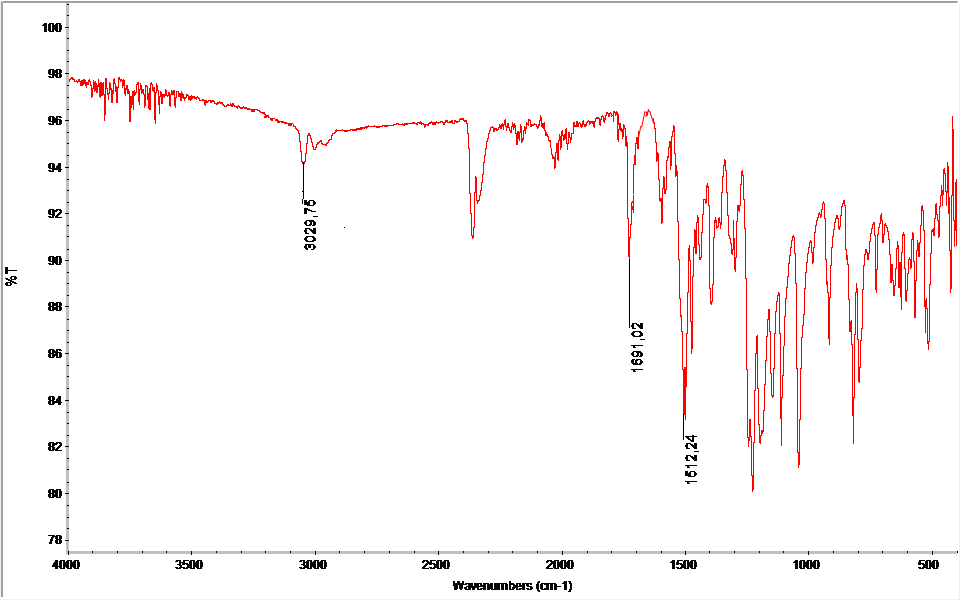


FT-IR spectrum of compound **1b**


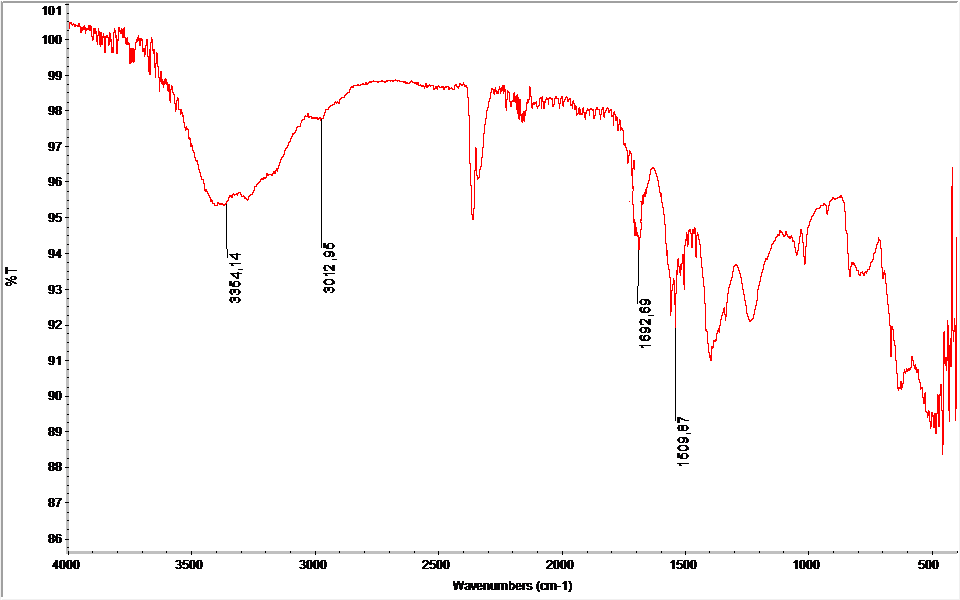


FT-IR spectrum of compound **1c**

**
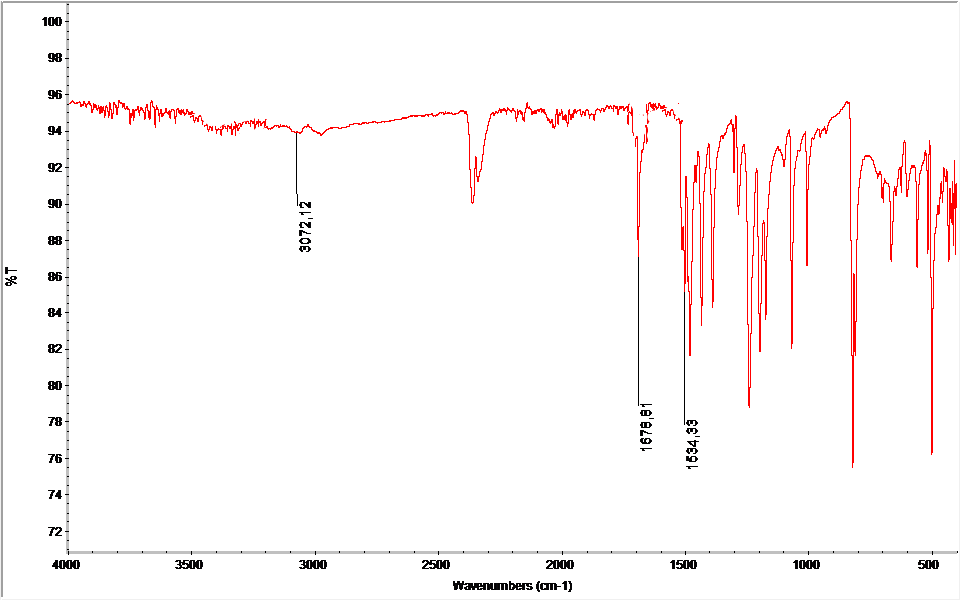
**

FT-IR spectrum of compound **1d**

**
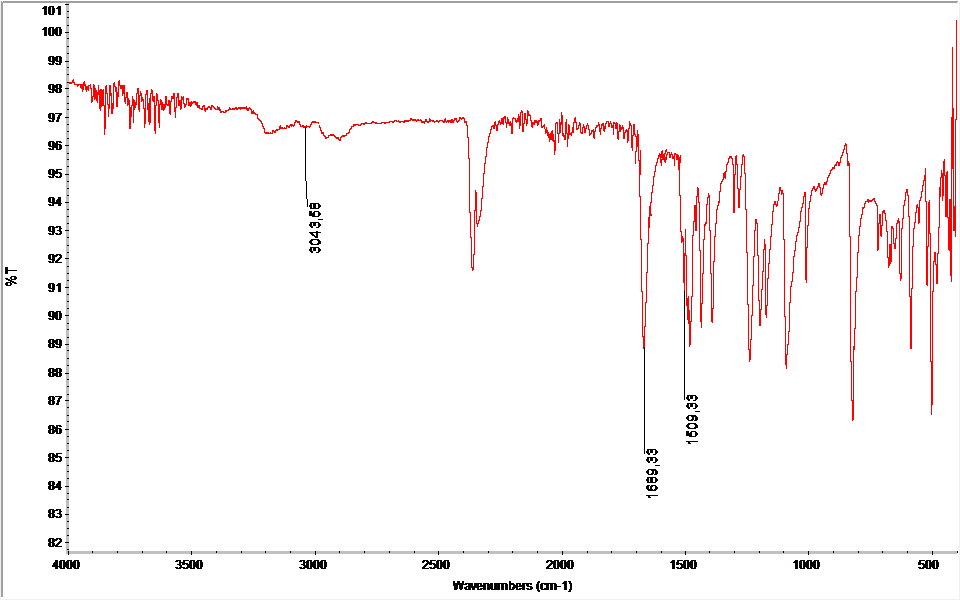
**

FT-IR spectrum of compound **1e**

**
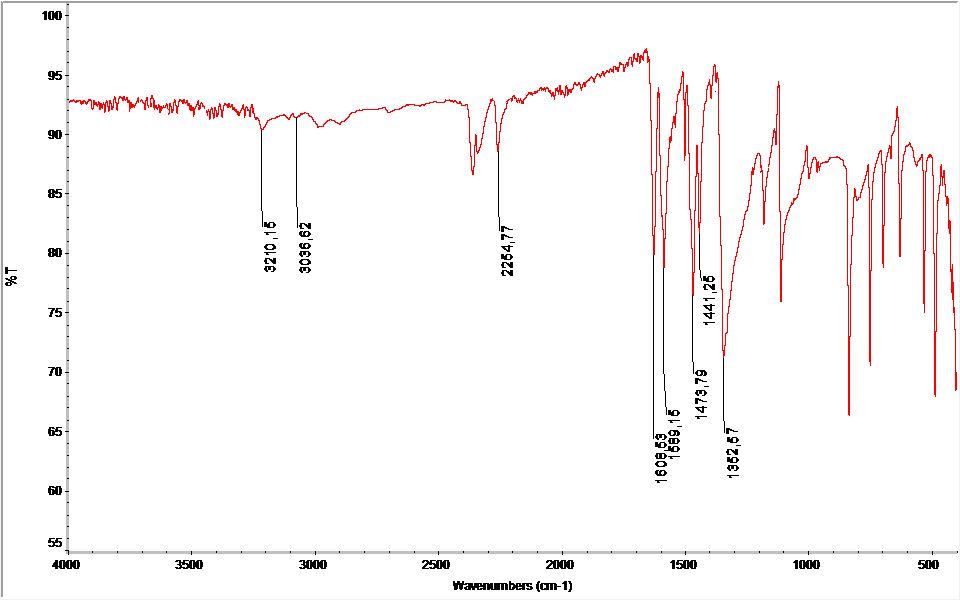
**

FT-IR spectrum of compound **2a**

**
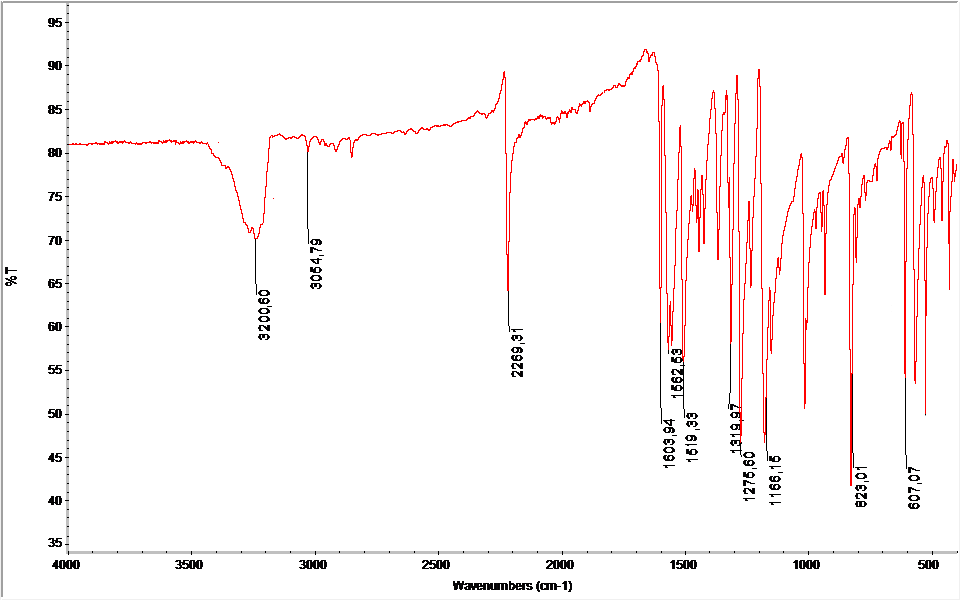
**

FT-IR spectrum of compound **2b**

**
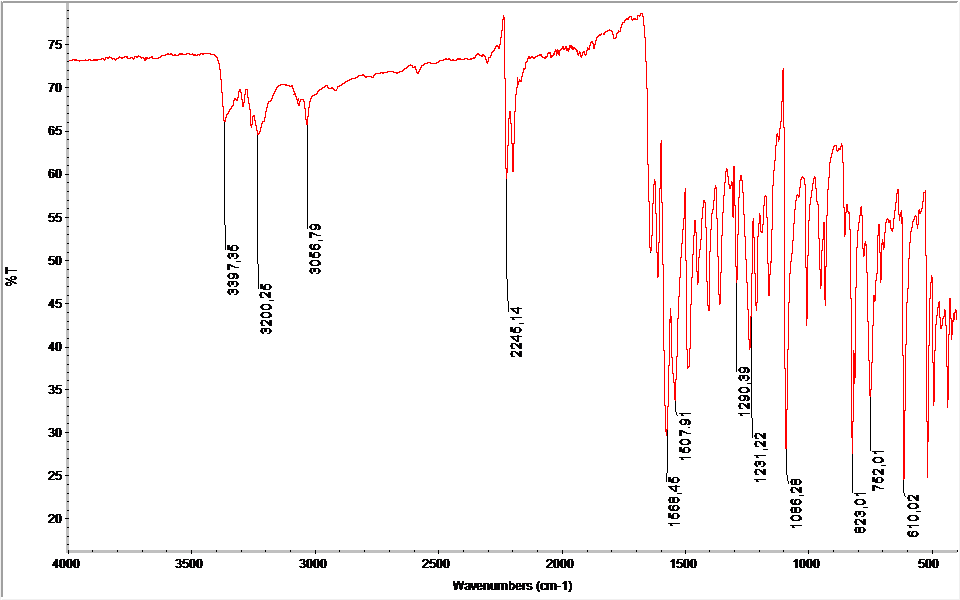
**

FT-IR spectrum of compound **2c**

**
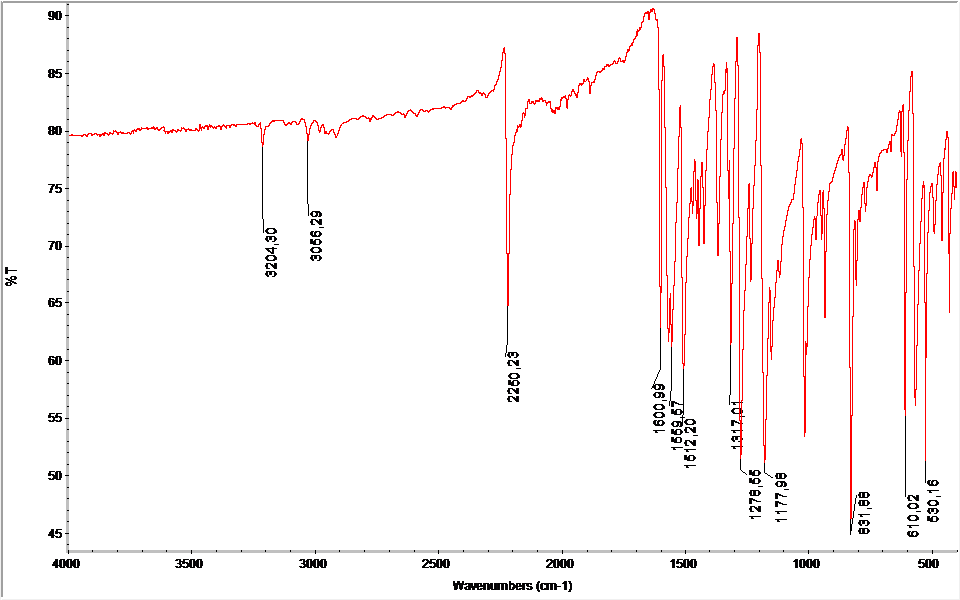
**

FT-IR spectrum of compound **2d**

**
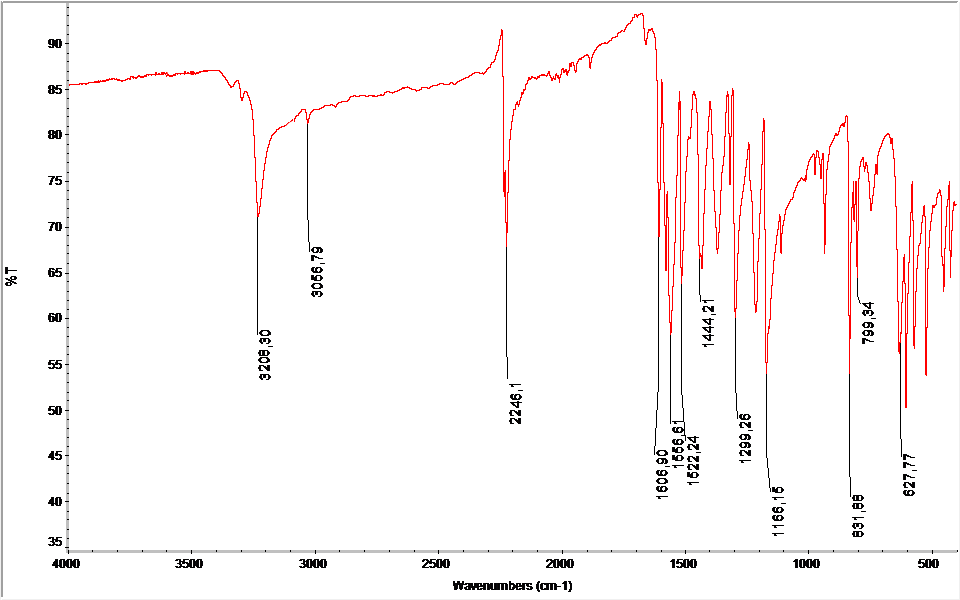
**

FT-IR spectrum of compound **2e**

**V. Table S1. Elemental Analysis Results of Compounds**

| **Sample** | **Weight (mg)** | **%C** | **%H** | **%N** | **%S** | **%Br** | **%Cl** | **C/N** |
| --- | --- | --- | --- | --- | --- | --- | --- | --- |
| **1a** | 1.008 | 53.75 | 4.08 | 22.70 | - | - | - | 2.37 |
| **1b** | 1.002 | 63.77 | 6.14 | 17.09 |  |  |  | 3.73 |
| **1c** | 1.009 | 60.91 | 5.09 | 19.31 | - | - | - | 3.15 |
| **1d** | 1.001 | 47.29 | 3.59 | 14.97 | - | 28.47 | - | 3.16 |
| **1e** | 1.005 | 56.17 | 4.27 | 17.79 | - | - | 15.00 | 3.16 |
| **2a** | 1.003 | 51.66 | 3.08 | 25.72 | 9.79 | - | - | 2.00 |
| **2b** | 1.008 | 59.20 | 4.63 | 21.49 | 9.80 | - | - | 2.76 |
| **2c** | 1.005 | 56.67 | 3.72 | 23.52 | 10.74 | - | - | 2.41 |
| **2d** | 1.009 | 46.82 | 2.79 | 19.40 | 8.86 | 22.14 | - | 2.41 |
| **2e** | 1.003 | 53.39 | 3.17 | 22.15 | 10.12 | - | 11.19 | 2.41 |

**VI. Table S2. ADME Pharmacokinetics and Druglikeness properites of compounds**

| **Compounds** | **GI absorption** | **BBB permeant** | **Lipinski** |
| --- | --- | --- | --- |
| 1a | High | No | Yes; 0 violation |
| 1b | High | Yes | Yes; 0 violation |
| 1c | High | Yes | Yes; 0 violation |
| 1d | High | Yes | Yes; 0 violation |
| 1e | High | Yes | Yes; 0 violation |
| 2a | Low | No | Yes; 0 violation |
| 2b | High | No | Yes; 0 violation |
| 2c | High | No | Yes; 0 violation |
| 2d | High | No | Yes; 0 violation |
| 2e | High | No | Yes; 0 violation |

**VII. Table S3. Bioavailability radar analysis and The BOILED-Egg ADME diagram of the compounds 1a-1e and 2a-2e WLOGP vs. TPSA**

| **Compound** | **Radar** | **Boiled Egg**  **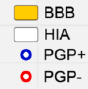** |
| --- | --- | --- |
| **1a** | **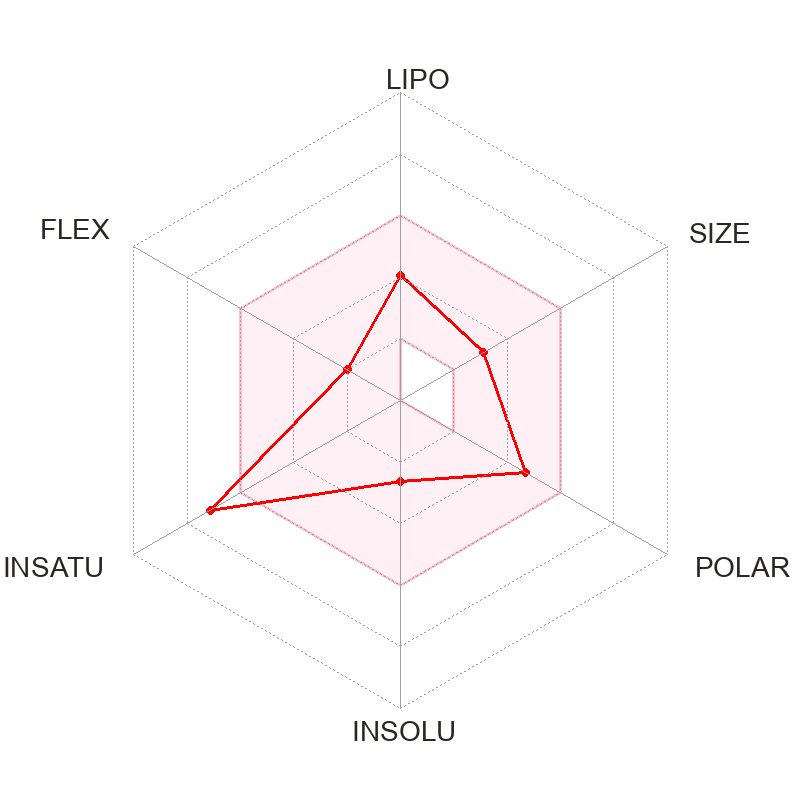** | **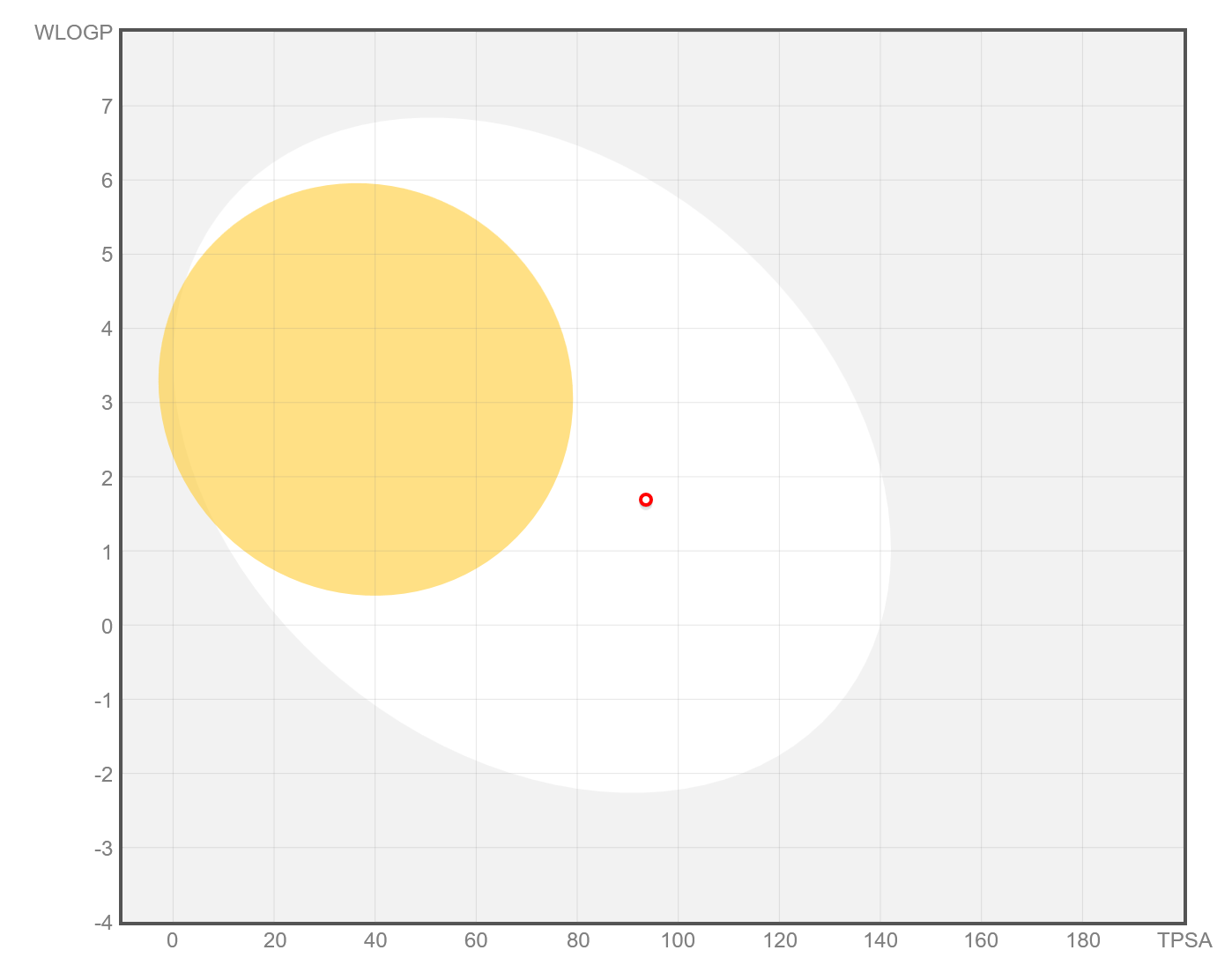** |
| **1b** | **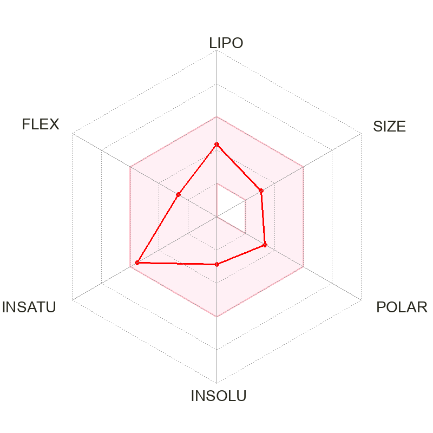** | **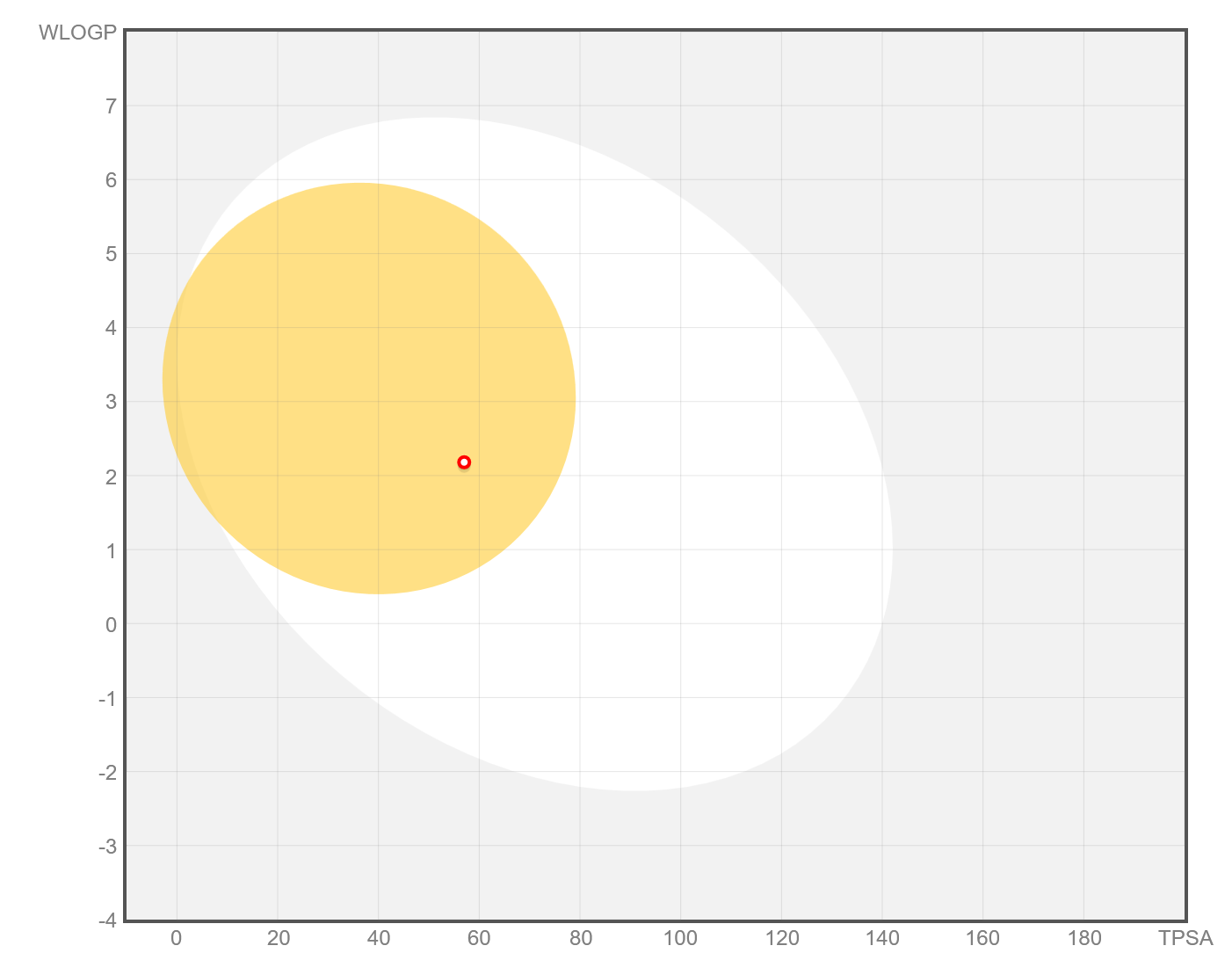** |
| **1c** | **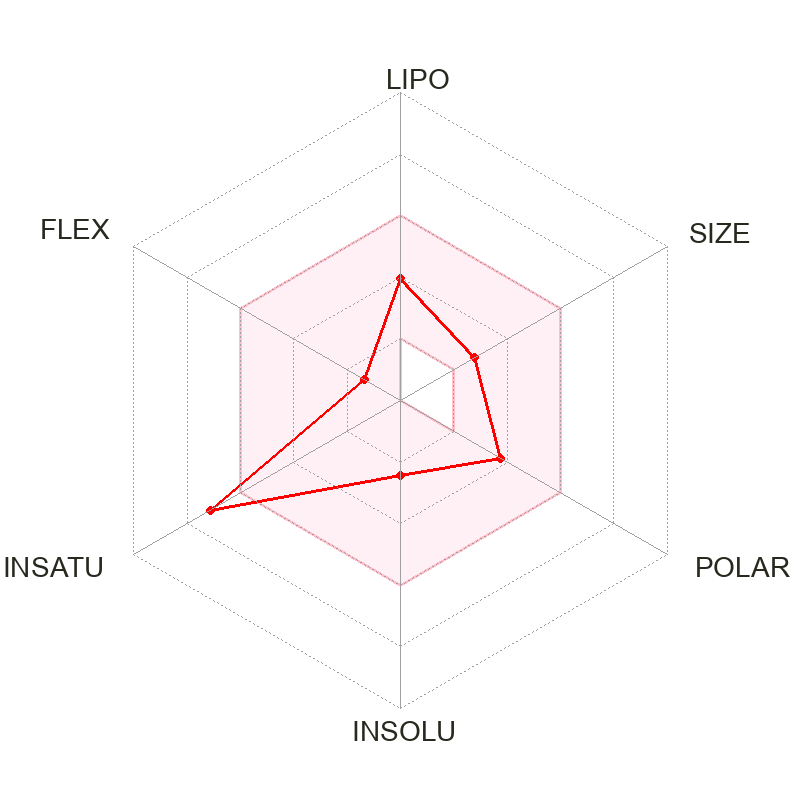** | **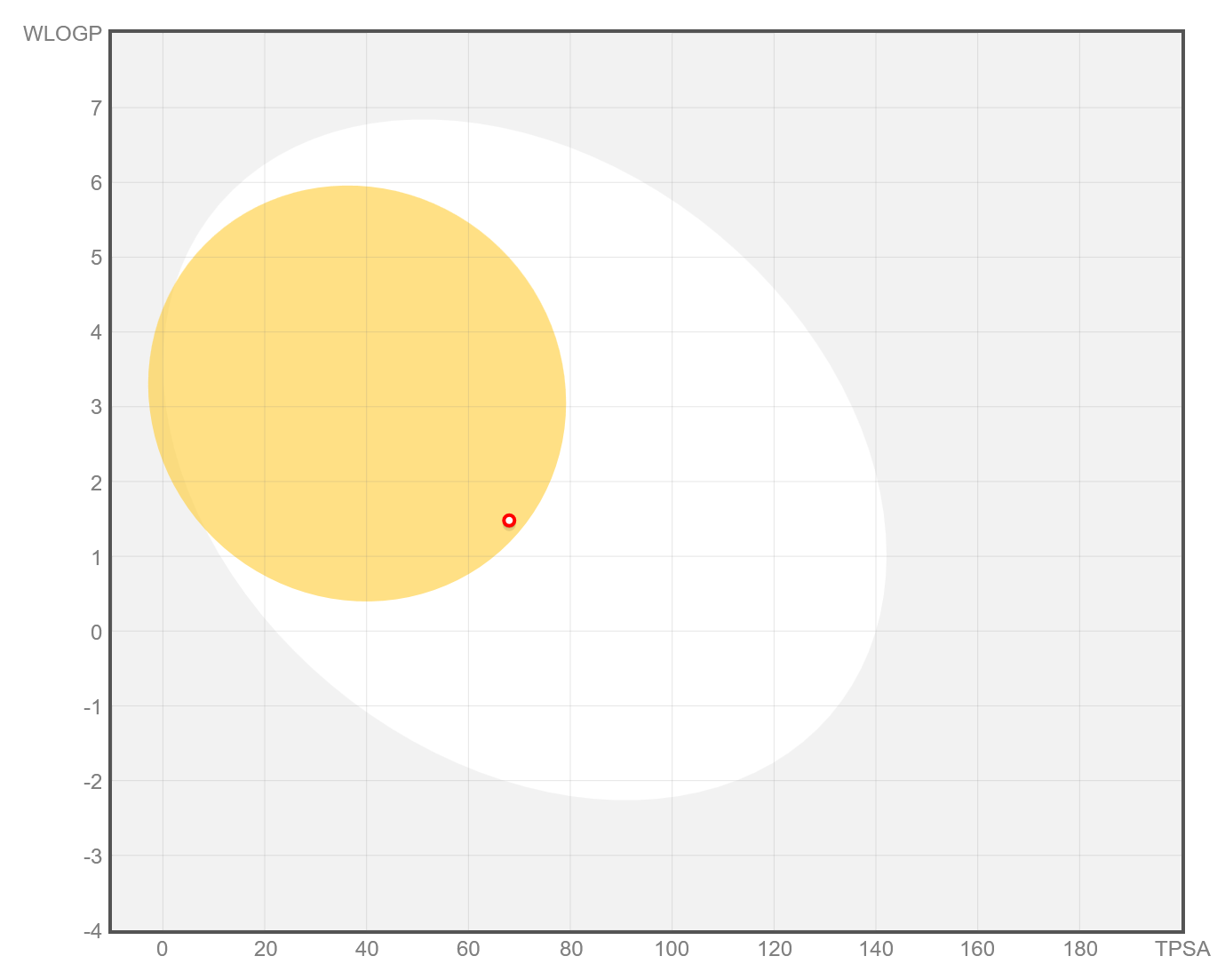** |
| **1d** | **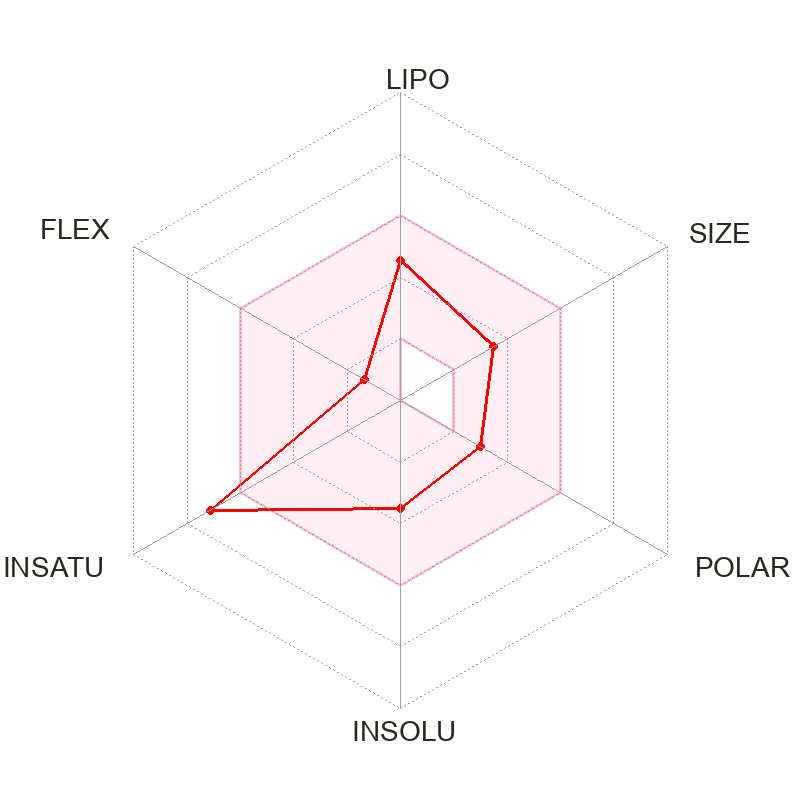** | **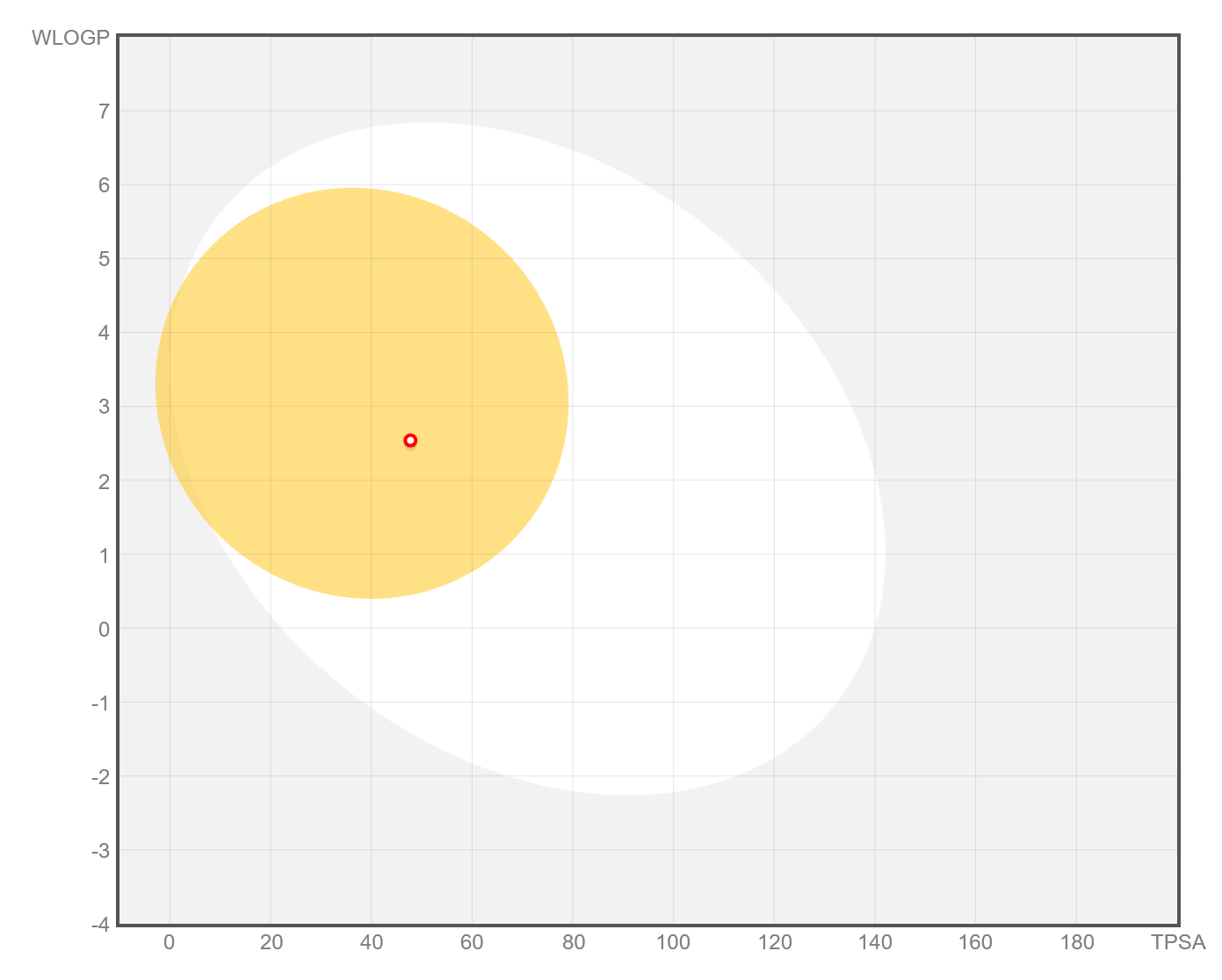** |
| **1e** | **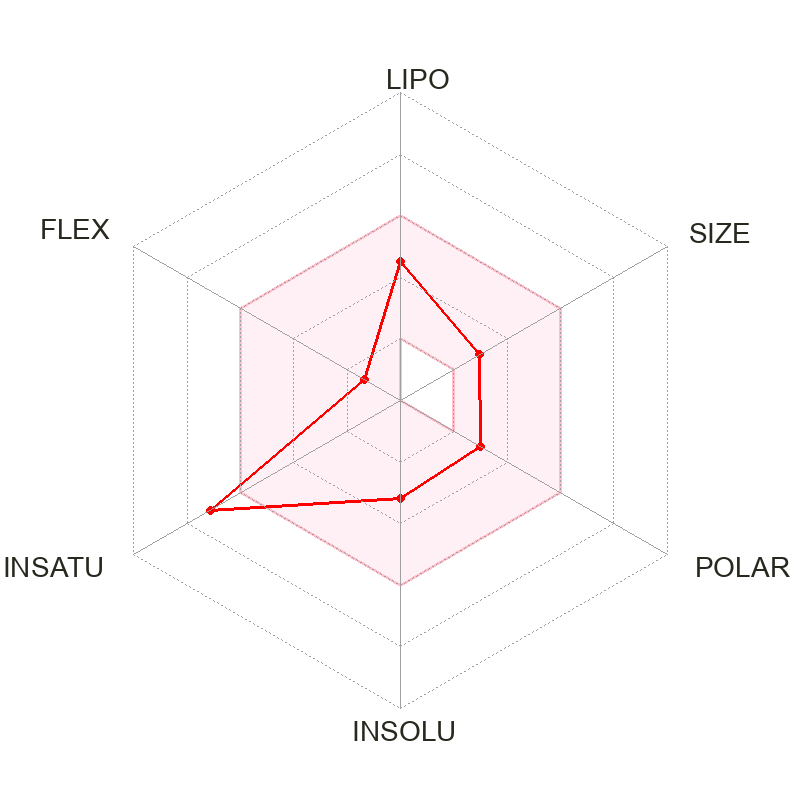** | **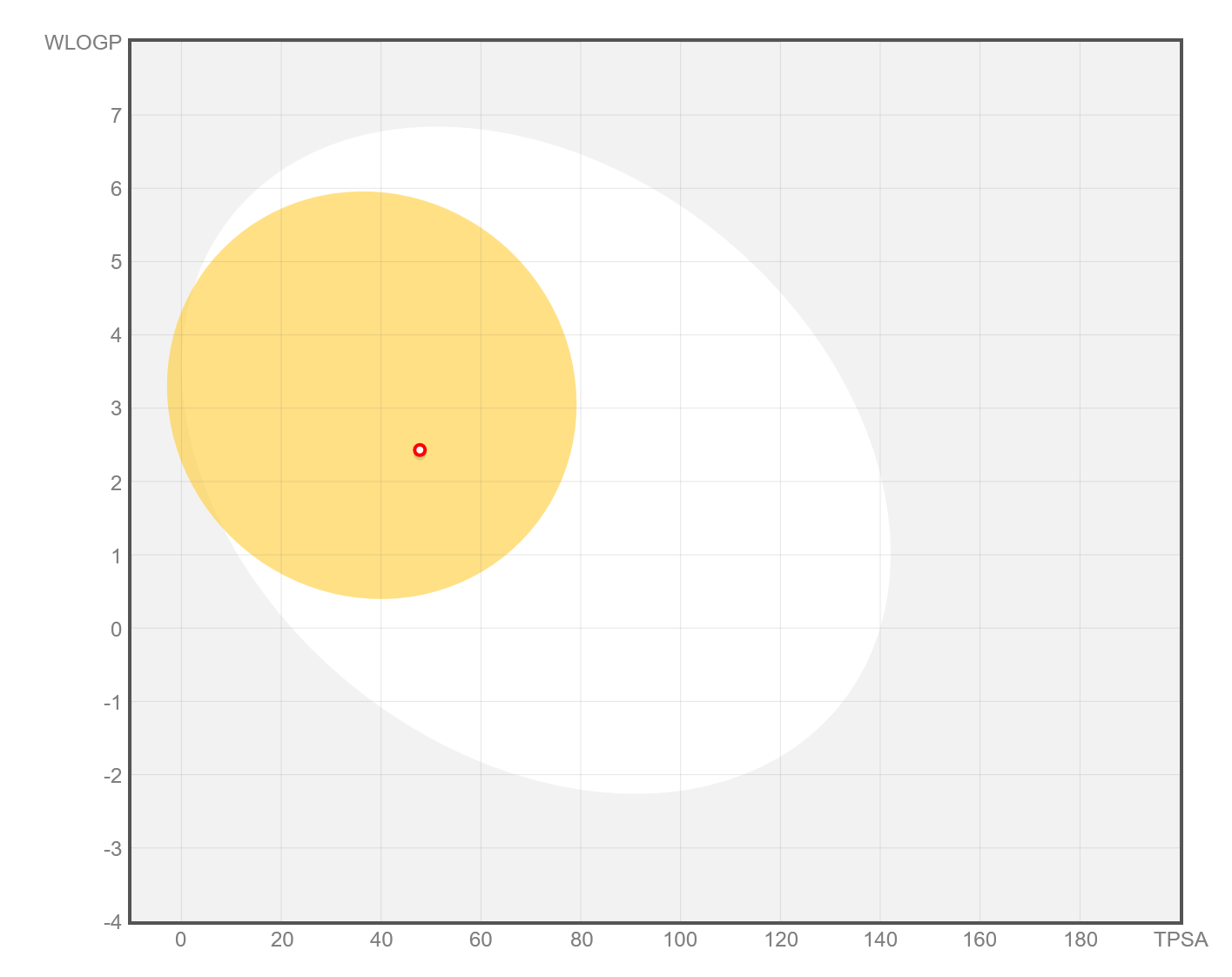** |
| **2a** | **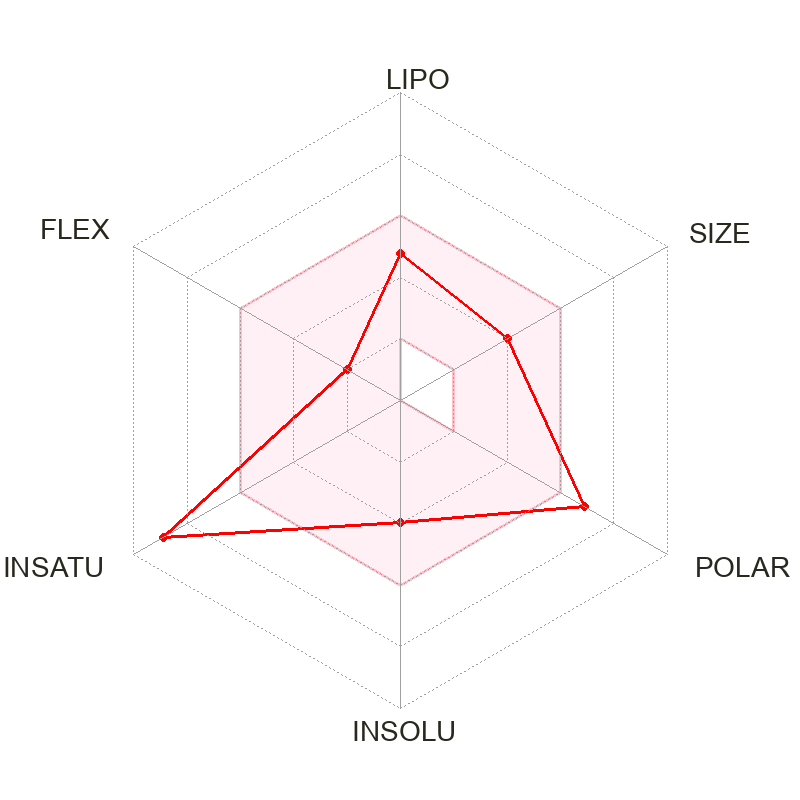** | **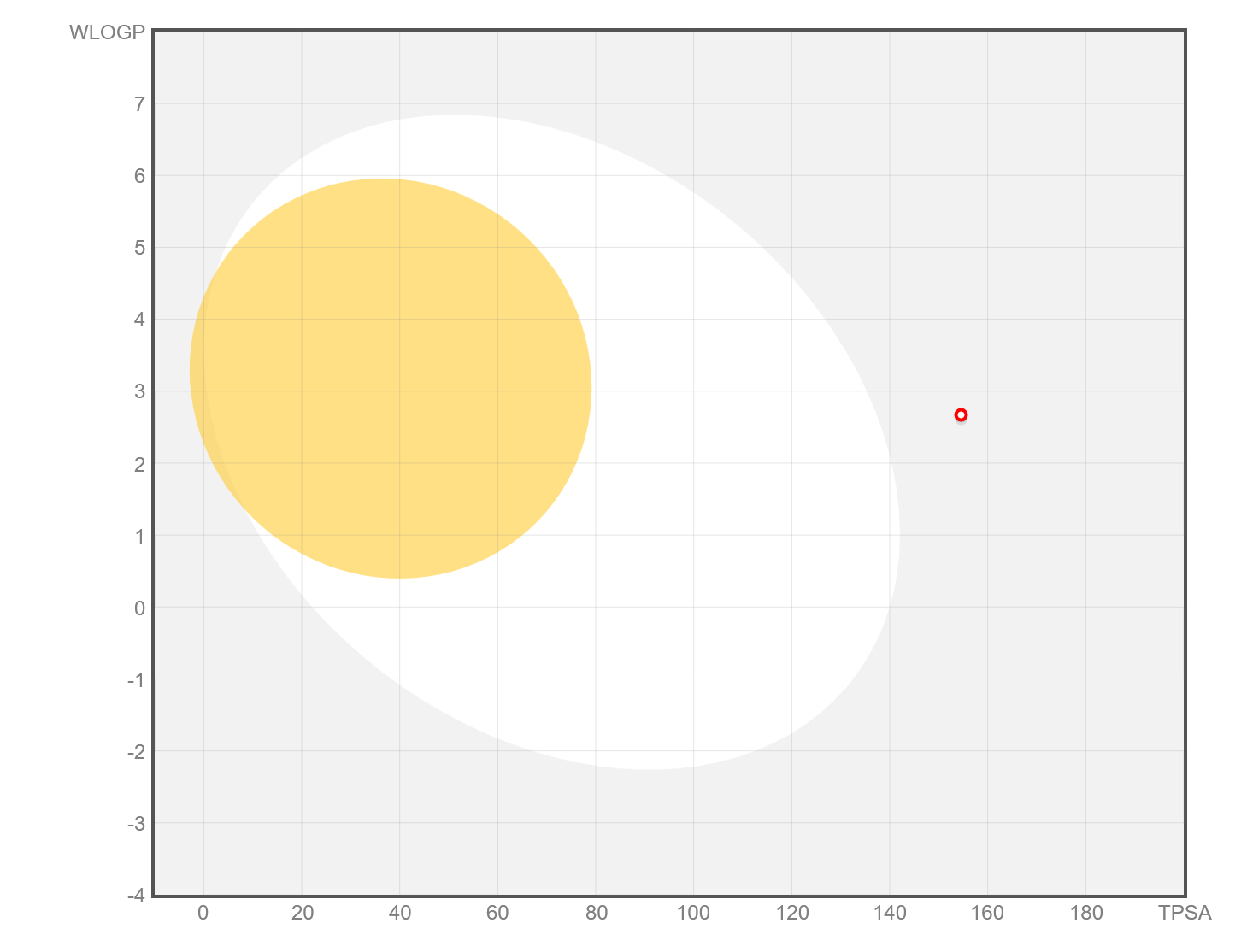** |
| **2b** | **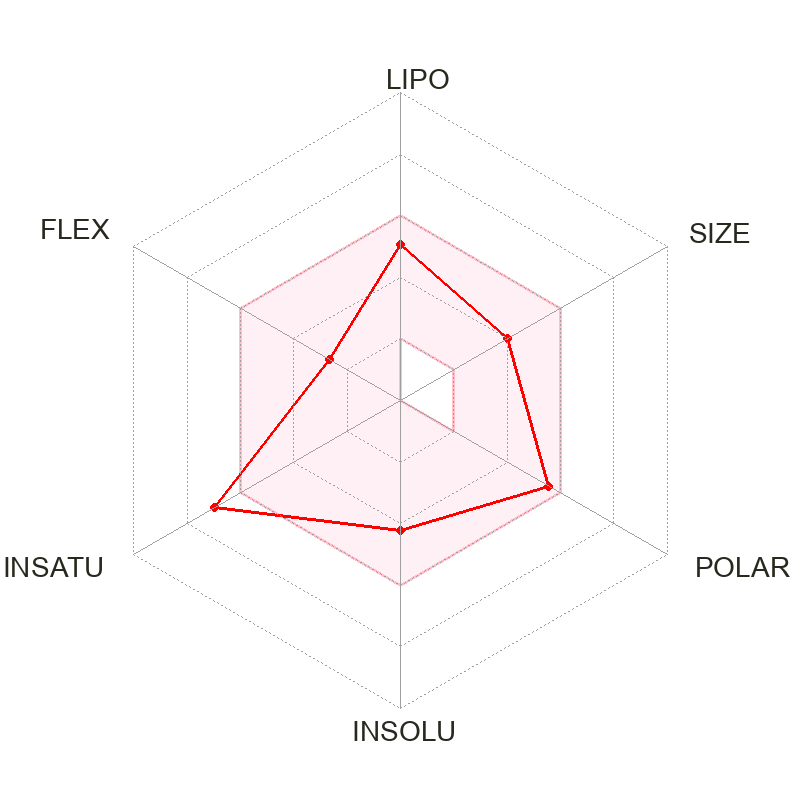** | **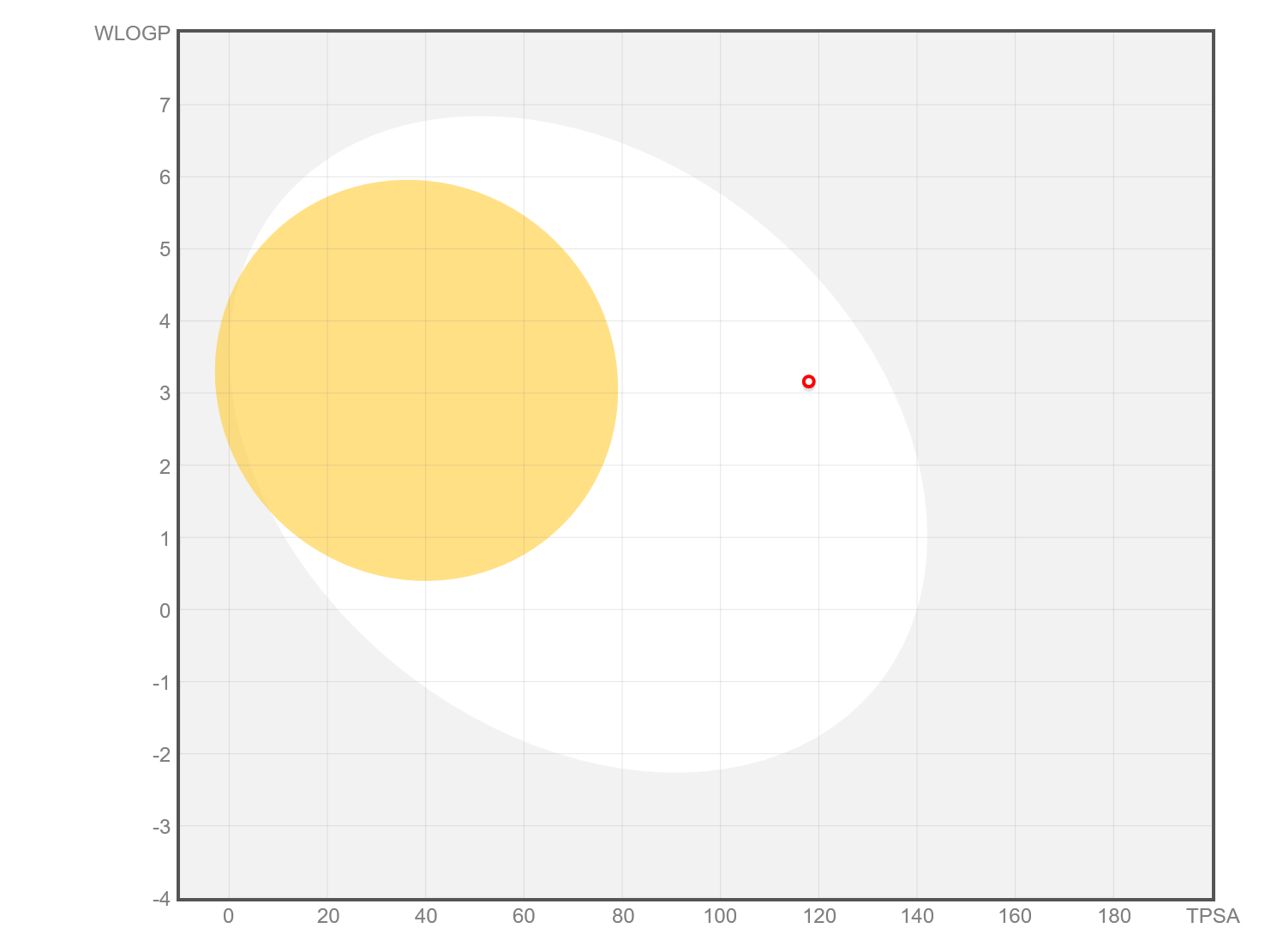** |
| **2c** | **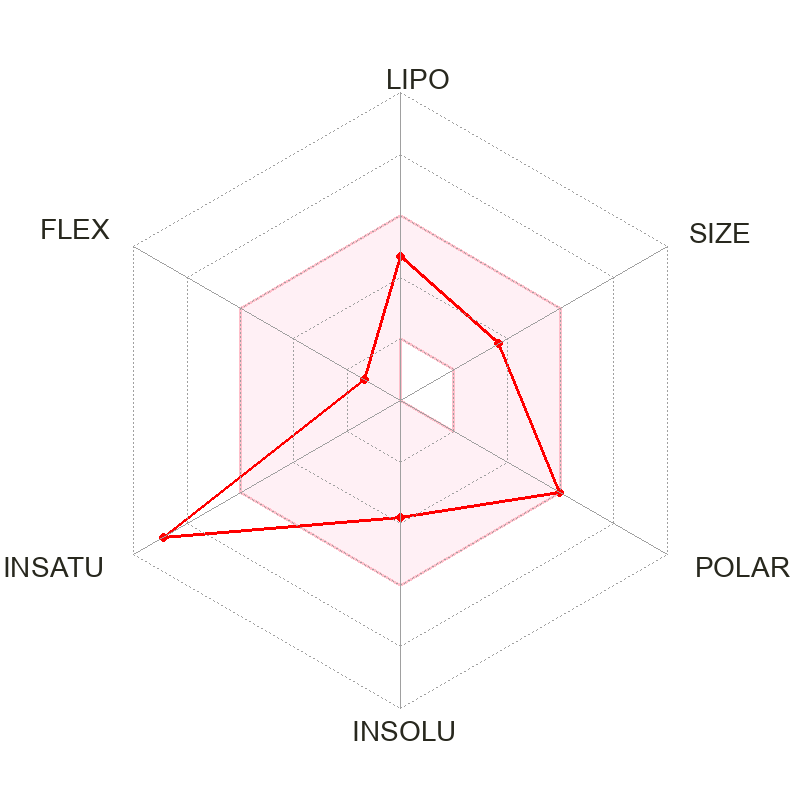** | **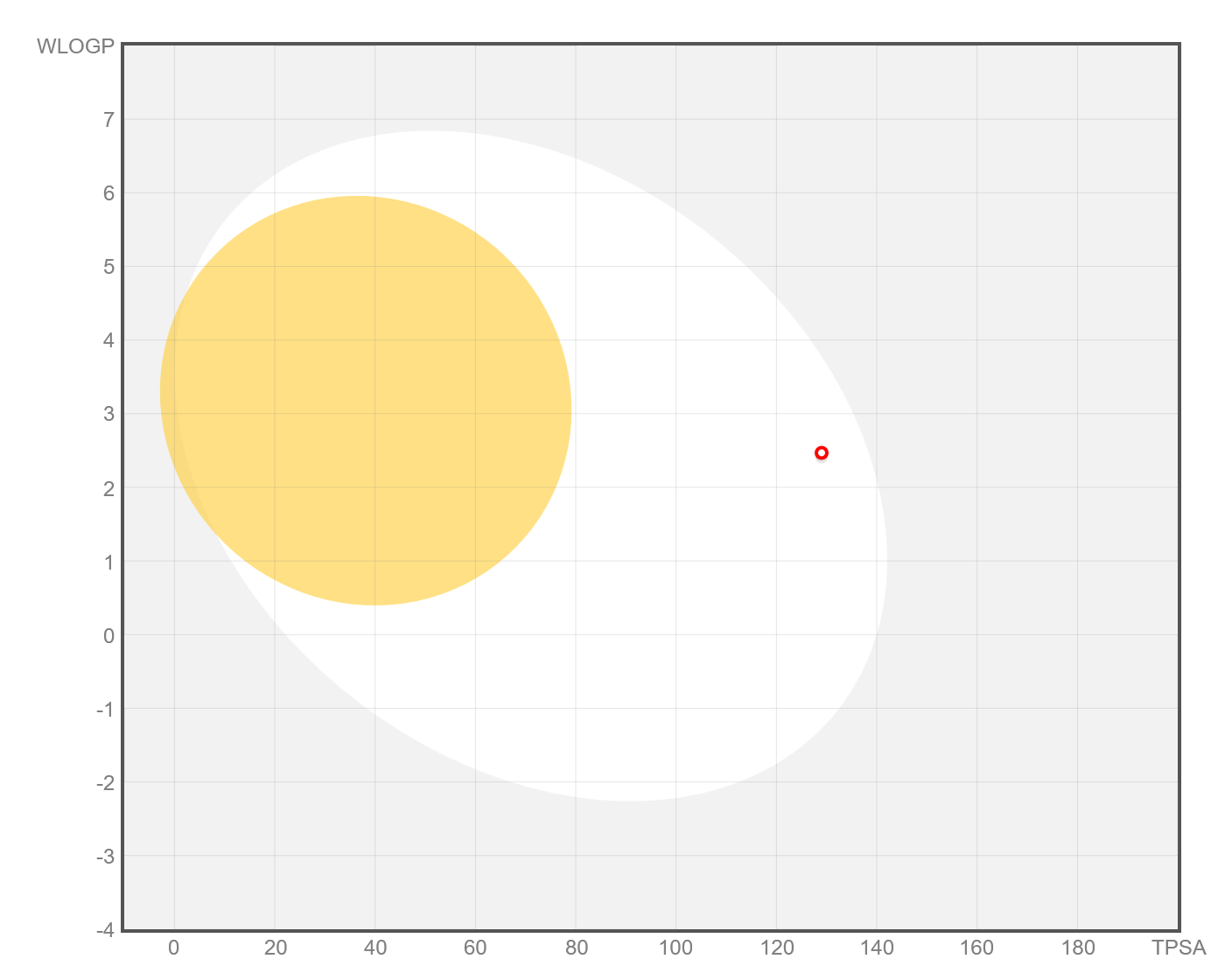** |
| **2d** | **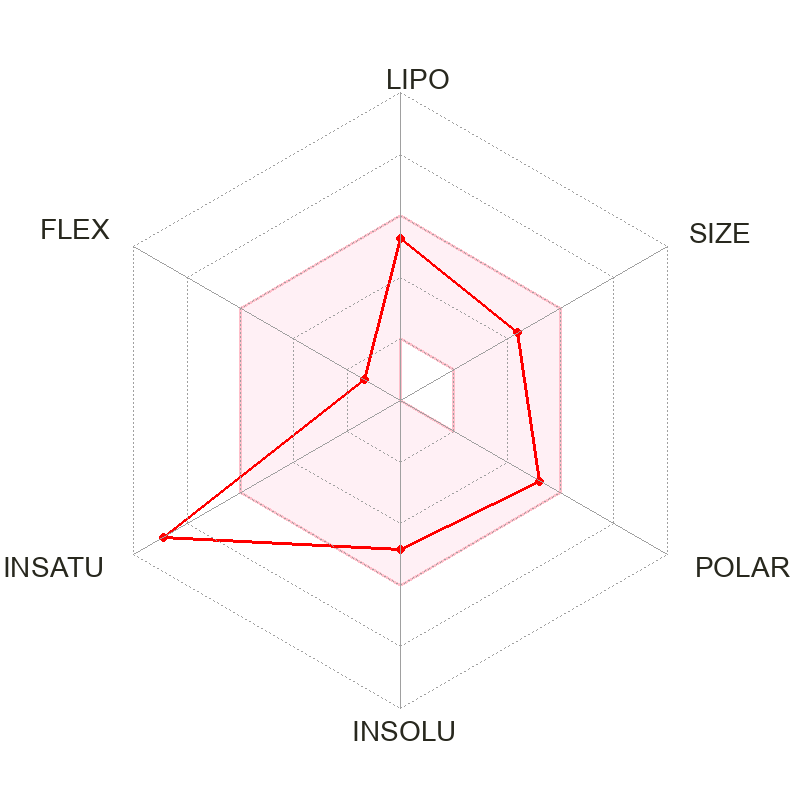** | **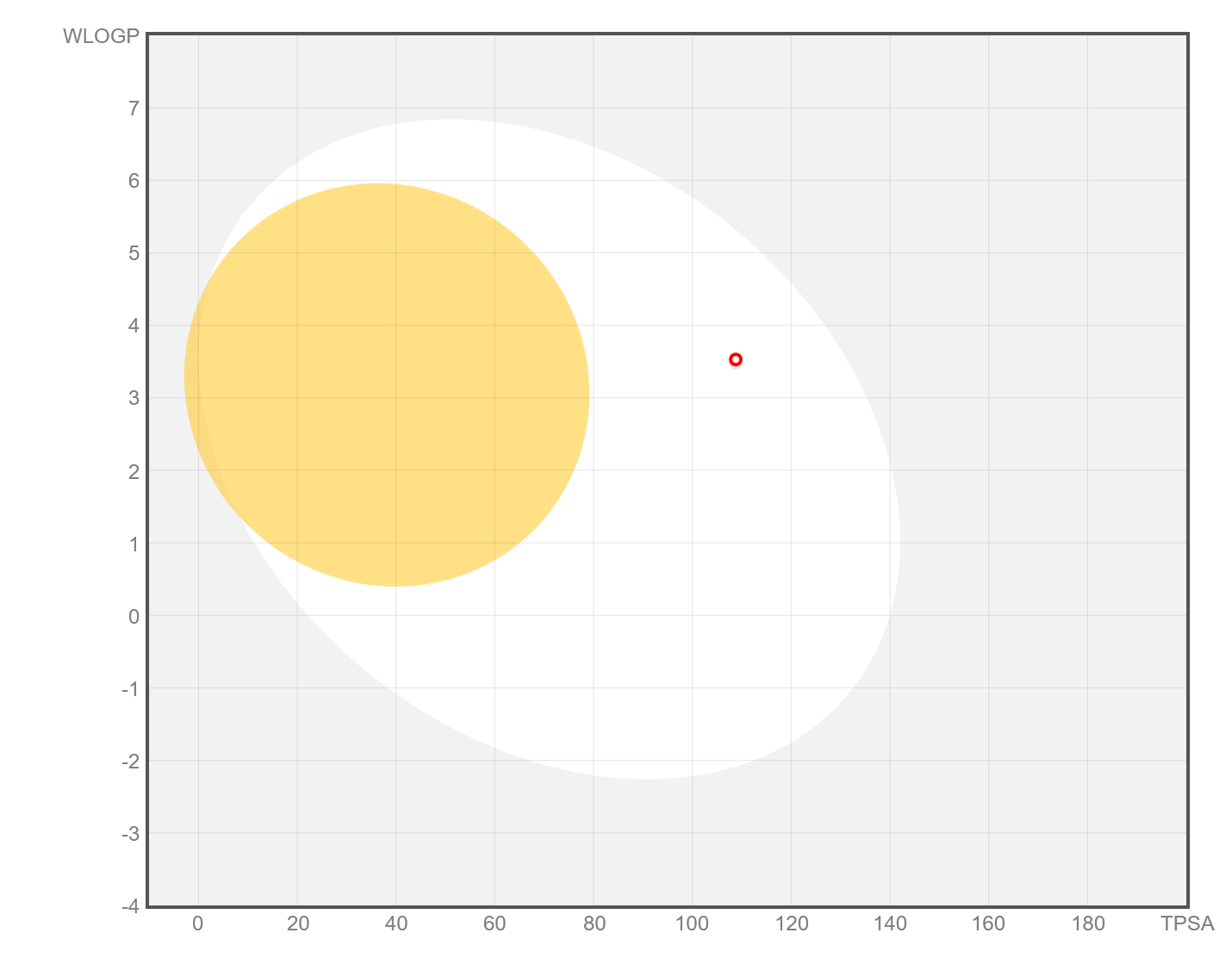** |
| **2e** | **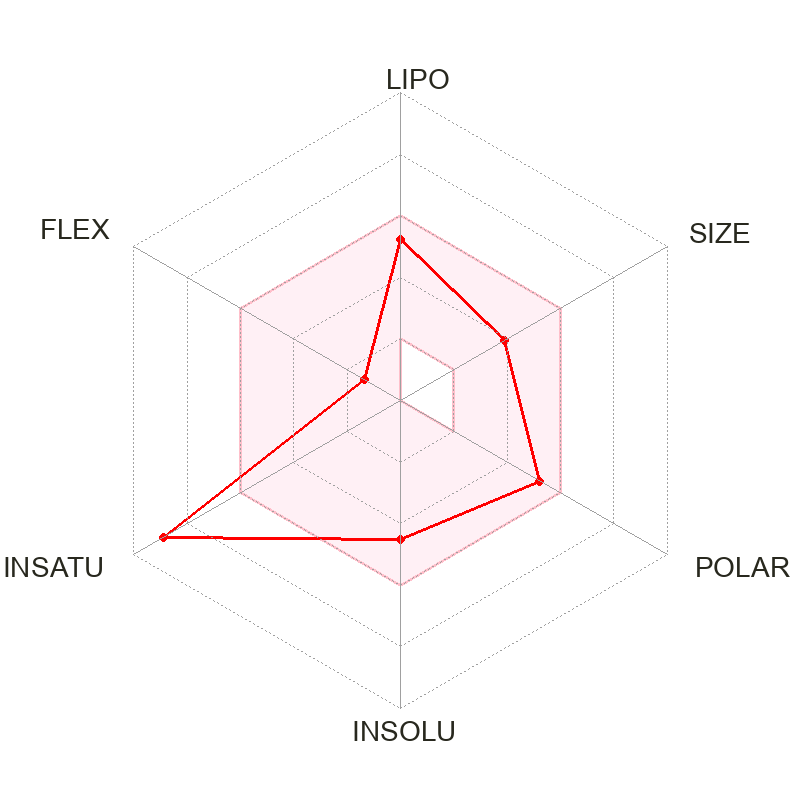** | **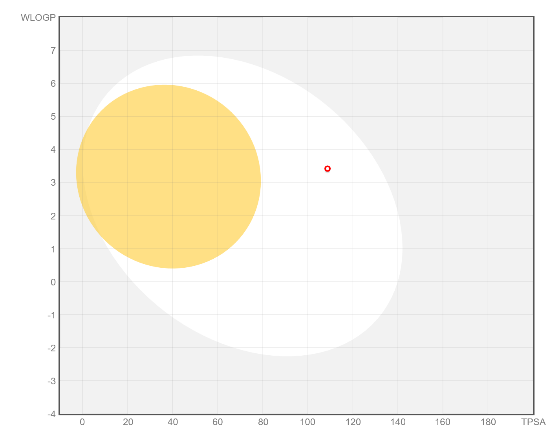** |
